# Directing the Mobility of Guest Molecules in Nanoporous Materials by Linearly Polarized Light

**DOI:** 10.1002/advs.202503500

**Published:** 2025-11-27

**Authors:** Taher Al Najjar, Chun Li, Yunzhe Jiang, Anna Mauri, Modan Liu, Abhinav Chandresh, Anemar Bruno Kanj, Dragos Mutruc, Wolfgang Wenzel, Stefan Hecht, Mariana Kozlowska, Lars Heinke

**Affiliations:** ^1^ Institute of Chemistry and Biochemistry Freie Universität Berlin 14195 Berlin Germany; ^2^ Institute of Functional Interfaces (IFG) Karlsruhe Institute of Technology (KIT) 76131 Karlsruhe Germany; ^3^ Institute of Nanotechnology (INT) Karlsruhe Institute of Technology (KIT) 76131 Karlsruhe Germany; ^4^ Department of Chemistry and Center for the Science of Materials Berlin Humboldt‐Universität zu Berlin 12489 Berlin Germany

**Keywords:** azobenzene, ion mobility, linearly polarized light, surmof

## Abstract

For the advancements of photoresponsive materials with tunable properties, the usage of multidimensional signals is desired. Using the polarization of the light in addition to the wavelength represents a further parameter to control the materials properties. Here, the first‐time dynamic and reversible manipulation of the guest‐host properties of a nanoporous material by linearly polarized light (LPL) is reported. The material is based on a metal–organic framework (MOF) with photoresponsive azobenzene side groups covalently connected to the MOF structure. The azobenzene moieties are reversibly reoriented by LPL, making the MOF structure and, thus, the pores anisotropic. As a result, the mobility of the guest molecules in the pores of the initially isotropic material becomes anisotropic, which can be dynamically controlled by the light polarization. The experiments by impedance spectroscopy are supported by molecular dynamics (MD) simulations. The study shows that the light polarization can be a further parameter to modify the material properties, allowing a more complex and more refined level of control for smart materials.

## Graphical abstract

The polarization of light is introduced as a further parameter to dynamically and reversibly control the properties of a photoresponsive nanoporous material. It was used to control the mobility of the guest molecules in the pores of a metal–organic framework. The metal–organic framework contains azobenzene side groups which are reoriented by linearly polarized light, creating anisotropic pores.

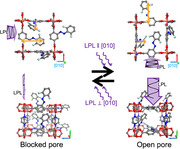

## 1 Introduction

The ability of molecules to undergo (directional) movement is fundamental for many processes in nature.^[^ ^1^, ^2^, ^3^, ^4^, ^5^ ^]^ The deliberate control of such molecular mobility on the nanoscale is a key feature for designing artificial smart materials and may open fundamentally new applications. Especially for advanced functions, a dynamic and intentional remote control by an external stimulus is desired. Using light as external stimuli is thus highly attractive as it allows for a fast and reversible modification of the material properties.^[^ ^6^, ^7^ ^]^ To this end, photochromic molecules can be incorporated in various materials, resulting in light‐responsive matters.^[^ ^8^, ^9^ ^]^ Based on this general approach, remote‐control over the mobility of molecules and ions inside the cavities of various porous materials has been demonstrated. This comprises the dynamic control of: i) diffusion and transport of molecules in (and through) nanopores,^[^ ^10^, ^11^, ^12^, ^13^, ^14^, ^15^, ^16^ ^]^ ii) proton conduction in nanopores,^[^ ^17^, ^18^, ^19^ ^]^ and iii) passage through bio‐pores.^[^ ^20^, ^21^ ^]^ All these studies switch between an on‐ and off‐state without further control over the orientation. Importantly, the direction of the molecular motion has not been controlled externally so far. Clearly, the deliberate control over alignment will enable further degree of control and may take the field of photoswitchable porous materials to the next level.

A particularly popular photochromic molecule with many applications in various solid materials is azobenzene.^[^ ^22^, ^23^ ^]^ Azobenzene undergoes light‐induced isomerization from the thermodynamically stable *trans* isomer to the metastable *cis* isomer by using light of a certain wavelength (often in the UV‐range). The back isomerization of *cis* azobenzene to the *trans* isomer can be induced by light of a different wavelength or by thermal relaxation. When azobenzene is functionalized by *ortho*‐positioned fluorine substituents, the *trans* → *cis* and the *cis* → *trans* isomerization can be induced by exciting the spectrally separated n–π* transition with green (ca. 530 nm) and blue (ca. 400 nm) light, respectively.^[^ ^24^, ^25^ ^]^ Such photochromic molecules have been incorporated in various kinds of materials, from polymers and liquid crystals over self‐assembled monolayers to metal–organic frameworks (MOFs).^[^ ^13^, ^17^, ^26^, ^27^, ^28^, ^29^, ^30^ ^]^ MOFs are a class of crystalline, nanoporous materials, which are made of inorganic metal nodes connected by organic ligand molecules.^[^ ^31^, ^32^ ^]^ In recent years, MOFs with azobenzene and other photochromic molecules have been explored for various aims, including photoswitchable control over adsorption^[^ ^33^ ^]^ and diffusion,^[^ ^34^ ^]^ membrane‐separation,^[^ ^10^, ^13^, ^35^ ^]^ as well as electronic^[^ ^36^, ^37^ ^]^ and ionic (proton)^[^ ^17^, ^18^ ^]^ conduction.

The dynamic control of the orientation of the photochromic molecules by light is a challenge. A very promising approach is the illumination of the material with linearly polarized light (LPL). There, it was found that the molecules dynamically reorient by repeated isomerization until the excited transition dipole moment is perpendicular to the light polarization, so that the excitation probability is minimized.^[^ ^38^ ^]^ That effect is known as Weigert effect and was extensively explored for azobenzene‐derivatives in polymer and liquid‐crystal films.^[^ ^39^, ^40^, ^41^ ^]^ The orientation of azobenzene molecules embedded in the pores of MOF films by using LPL was recently demonstrated.^[^ ^42^ ^]^ For a MOF film with fluorinated‐azobenzene‐side‐groups pendant to the linker molecules, it was shown that circularly polarized light (CPL) allows to reversibly enrich the enantiomers of the MOF‐linkers and induces chirality in the material.^[^ ^43^ ^]^ Using polarized light to control or direct the mobility of guest molecules in MOFs (or other porous materials) has not yet been reported to date.

**Figure 1:**
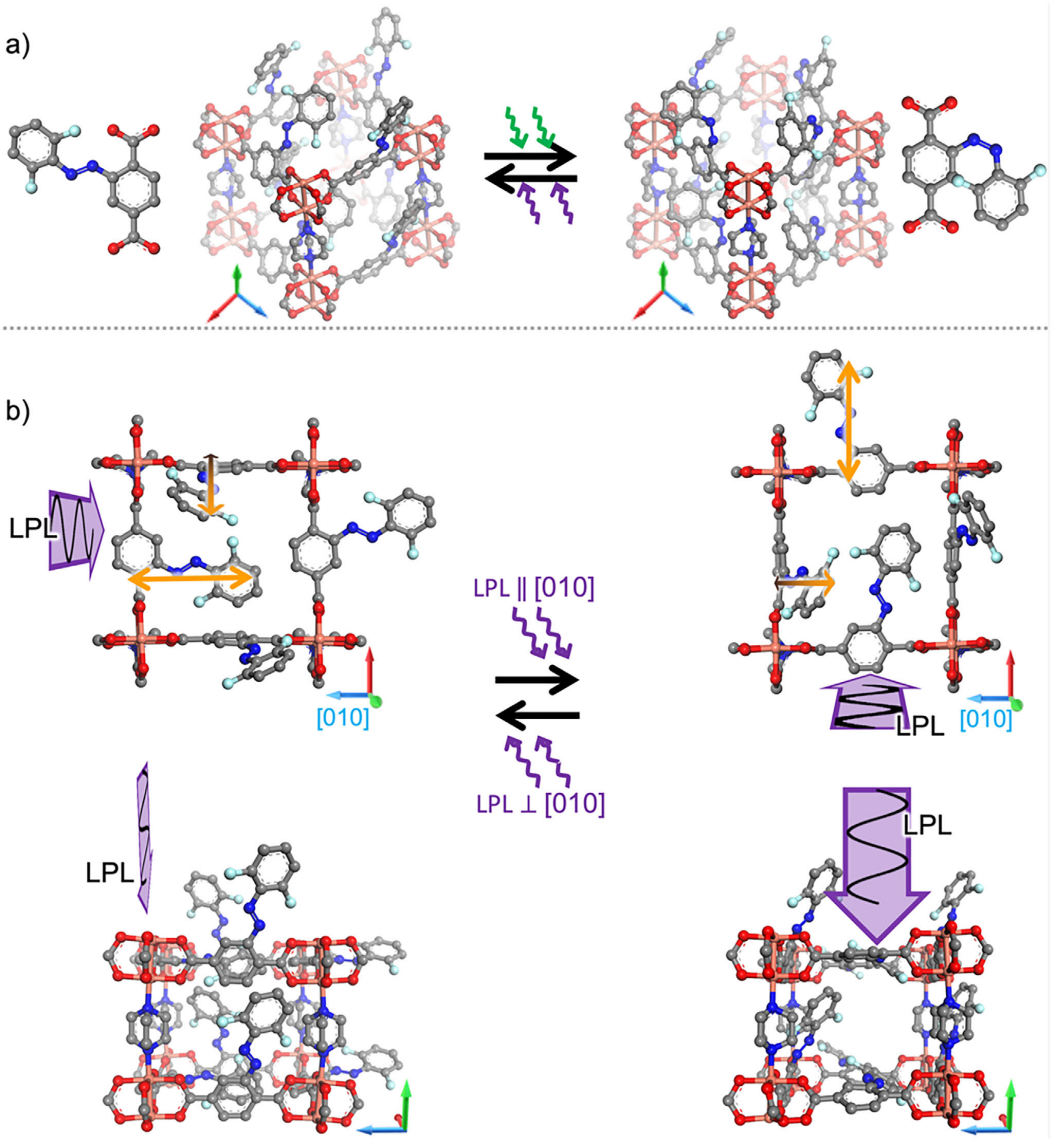
a) Photoswitchable Cu_2_(F_2_AzoBDC)_2_(dabco) SURMOF film in the *trans* state (upon violet light irradiation, left) and in the *cis* state (upon green light irradiation, right). The F_2_AzoBDC linker molecule in the *trans* and *cis* configuration is also shown. b) Sketch of the SURMOF with oriented *trans*‐azobenzene side groups as a result of the light polarization. Here, the propagation direction of the (violet) light beam corresponds to the [001] direction of the MOF crystal. The light polarization is parallel to the [100] direction (i.e., perpendicular to [010]) on the left‐hand side and parallel to the [010] direction on the right‐hand side. The structures are shown for the view along the [001] direction above and along the [100] direction in the bottom. (Apart from the different point of view, the structures in the bottom and above are identical.) The tripods indicate the crystal directions in the sketches: [100] ‐ red, [010] ‐ blue, and [001] ‐ green. The transition dipole moments (TDMs) of the azobenzene moiety (n–π*‐transition) are sketched by the orange arrows in the upper sketches.

Here, we present the dynamic and reversible control of the direction of the guest molecule motion in an otherwise isotropic material. This is based on light‐induced preferred orientation of fluorinated azobenzene side groups in a MOF film by LPL (**Figure** **1**). The MOF has a pillared‐layer structure of type Cu_2_(F_2_AzoBDC)_2_(dabco) and was grown in a layer‐by‐layer fashion as uniform thin film by using the surface‐mounted MOF (SURMOF) approach.^[^ ^44^, ^45^ ^]^ The material seems perfectly suited for this study, since a high material durability and very large fatigue resistance was already demonstrated in previous studies.^[^ ^13^, ^17^, ^43^, ^46^, ^47^, ^48^ ^]^ We show that by exciting the n–π* transition with LPL of 405 nm, the azobenzene side groups are predominantly oriented perpendicular to the plane of the light polarization, permitting opening of the MOF pore. The UV–vis and IR spectroscopy data indicate that the orientation ratio is 4.3 %. The mobility of guest molecules in the MOF pores, here 1‐butyl‐3‐methylimidazolium cations and bis(trifluoromethylsulfonyl)imide anions, i.e., [BMIM][TFSI], was explored by electrochemical impedance spectroscopy (EIS). The data show that the mobility of the molecules in the pores of the intrinsically isotropic material (i.e. isotropic in the (001) plane) is clearly larger in the direction perpendicular to the (light) polarization than parallel to the polarization. This modification is reversible and changes with the polarization. Additionally, molecular dynamic (MD) simulations provide insights on the anisotropic mobility on the molecules level. This is the first demonstration of the dynamic control of the direction of the mobility of guest molecules in a nanoporous material by polarized light.

## 2 Results and Discussion

**Figure 2:**
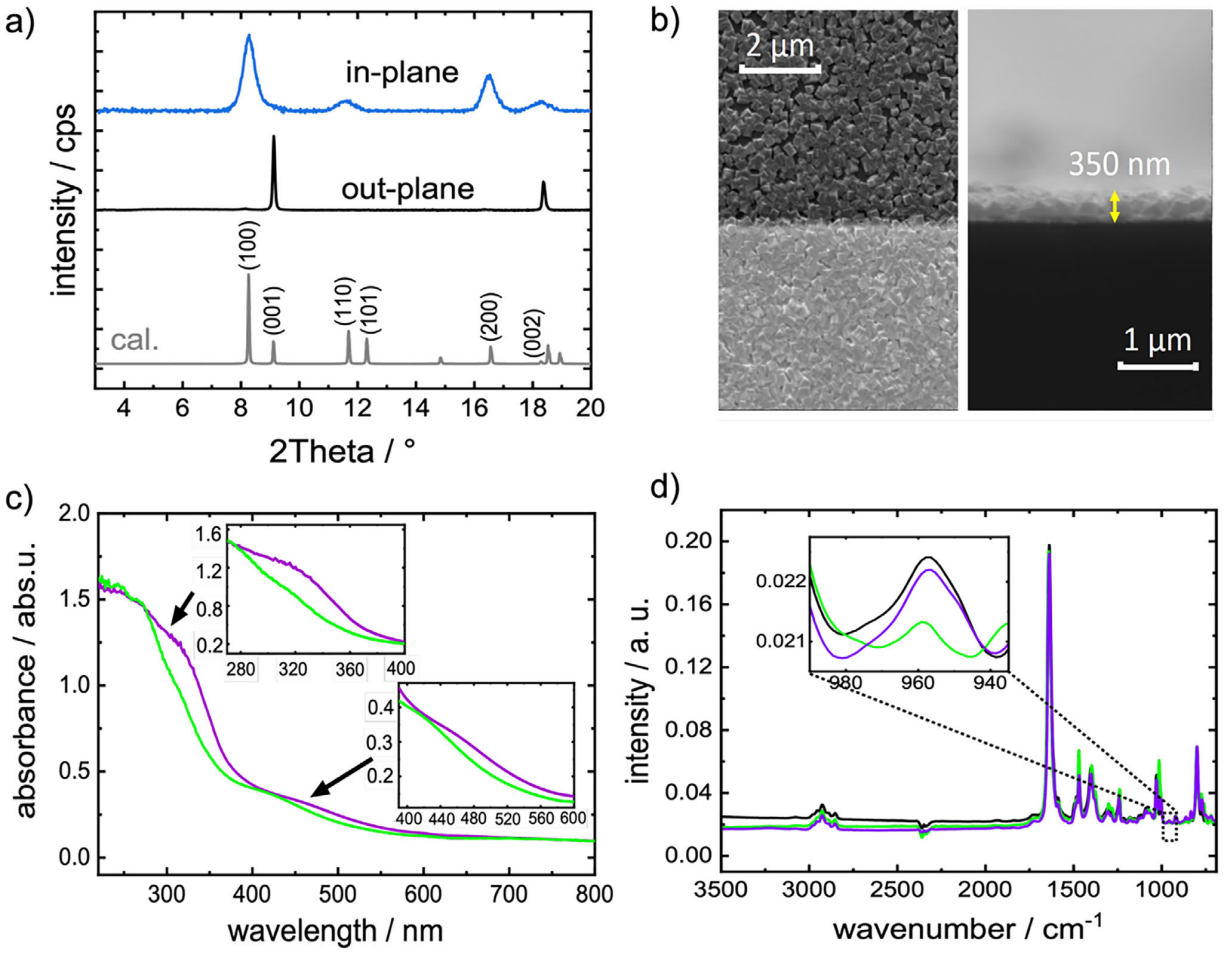
a) XRDs of the sample in out‐of‐plane geometry (black) and in‐plane geometry (blue) compared with the calculated diffractogram of the targeted structure (grey). The X‐ray wavelength is 0.154 nm. As reference, the XRD of the corresponding MOF powder is shown in Figure S14 (Supporting Information). b) SEM images of the sample. The top‐view (where the gold electrodes are visible as bright stripes) is shown on the left‐hand side, the side view of the broken sample is shown on the right‐hand side. More SEM images are shown in Figure S1 (Supporting Information). c) UV–vis spectra of the sample upon irradiation with green light (*cis*‐rich‐state, green) and subsequently upon irradiation with violet light (*trans*‐rich‐state, violet). The intensity changes of the π–π* at 320 nm and of the n–π* at 450–500 nm, respectively, are clearly visible. d) IR spectra of the sample in the pristine state (in black), upon green and violet light. Same color code as in c). The band at 960 cm^−1^ is assigned to the *trans*‐azobenzene^[^ ^13^ ^]^ and does not overlap with other absorption bands; thus, it can be used to quantify the isomer ratio. All spectra (c,d) are obtained upon irradiation with unpolarized light.

The MOF films were grown in a layer‐by‐layer fashion directly on the substrates. The MOF structure is a pillared‐layer structure of type Cu_2_(F_2_AzoBDC)_2_(dabco) where dabco stands for 1,4‐diazabicyclo[2.2.2]octane and F_2_AzoBDC for (*E*)‐2‐((2,6‐difluorophenyl)diazenyl)terephthalate. The X‐ray diffraction (XRD) data recorded in out‐of‐plane and in‐plane geometry, **Figure** **2**a, show that the film has the targeted MOF structure. The X‐ray diffraction data are in agreement with previous studies with this MOF structure.^[^ ^13^, ^17^, ^43^, ^46^, ^47^, ^48^, ^49^ ^]^ In addition, the XRD data show that the films are grown in a mainly oriented fashion, so that the [001] is in the direction of the surface normal. The SEM images, Figure 2b, show that the film has a rather homogenous morphology, and the film thickness is ≈350 nm. No indications for a significant percentage of areas or domains with different structures, morphology or film thickness were found in the SEM and XRD data, indicating the samples are grown as homogenous thin films. The UV–vis spectra of the sample, Figure 2c, show a clear reversible change of the absorption intensity at 320 nm (π–π* band) and at 450 nm (n–π* band) upon irradiation with unpolarized light of 405 and 530 nm, respectively. In detail, the π–π* band at 320 nm belongs to the *trans* isomer, the n–π* band at ≈400 nm belongs to the *cis* isomer and the n–π* band at ≈450–500 nm belongs to the *trans* isomer. This is in line with previous studies with SURMOFs of that structure^[^ ^13^, ^17^ ^]^ and with the studies of the photochromic molecule in solution,^[^ ^24^, ^25^ ^]^ as well as with our calculations (see Table S1, Supporting Information). The data indicate the 405 nm light‐induced *cis* → *trans* isomerization and the 530 nm light‐induced *trans* → *cis* isomerization. The switching yield was determined by using infrared spectroscopy, Figure 2d. The band at 960 cm^−1^ is assigned to the *trans* azobenzene isomer. From the decrease of the area upon irradiation (compared to the thermally relaxed, 100%‐*trans* sample), the isomer composition in the photostationary state (PSS) was determined to contain ≈89% *trans* and 11% *cis* isomer content upon 405 nm illumination, while upon illuminating with 530 nm 81% of *cis* and 19% of *trans* isomer are present in the PSS. This is also in agreement with previous studies.^[^ ^13^, ^17^, ^47^ ^]^

**Figure 3:**
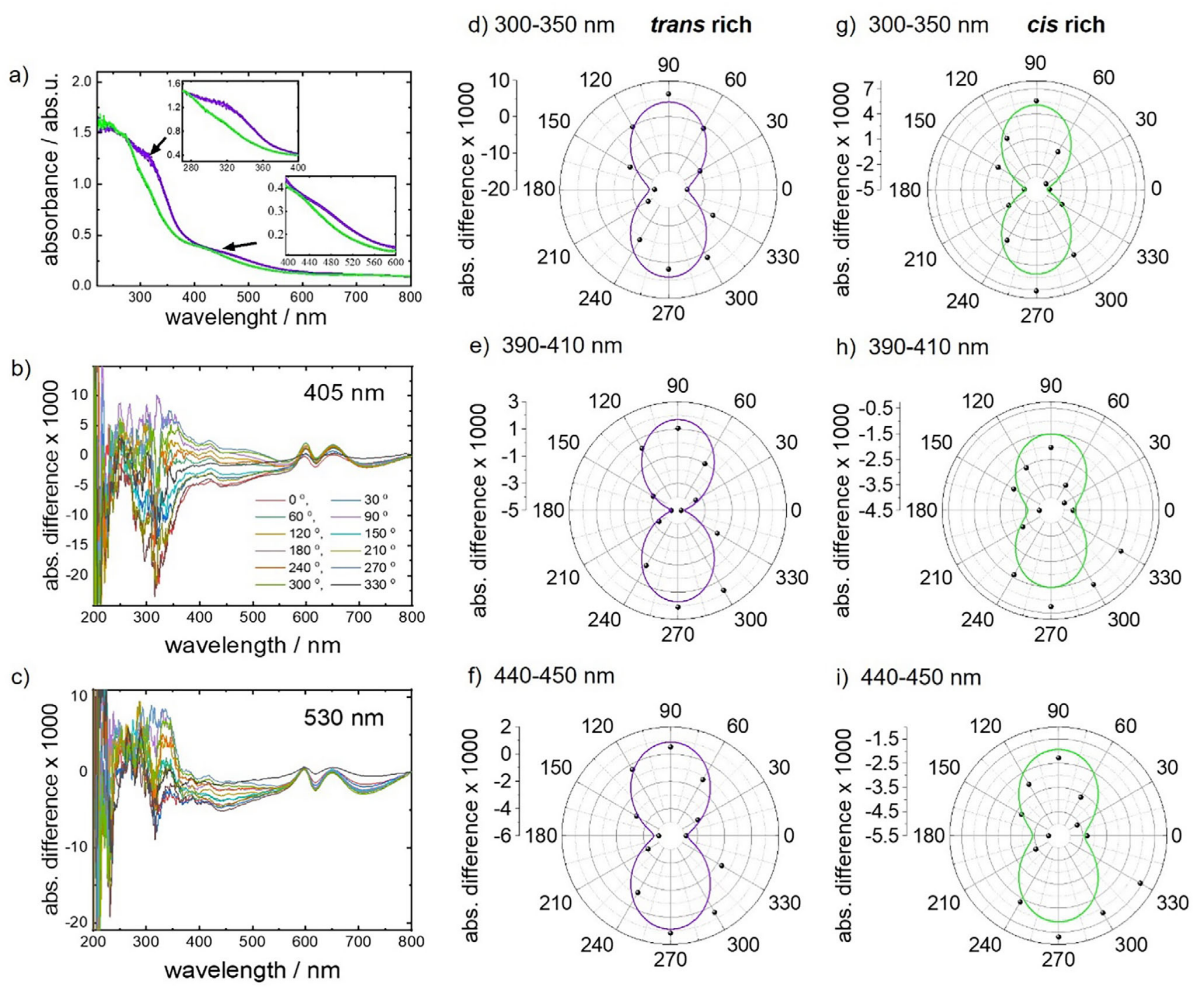
a) UV–vis spectra after irradiation with LPL of different angles with a wavelength of 405 nm (spectra in various shades of violet) and 530 nm (spectra in various shades of green). The insets show a magnification of the π–π* and n–π* absorption bands. b) UV–vis differential spectra upon 405 nm LPL irradiation with the spectrum upon unpolarized light irradiation subtracted as a baseline. c) UV–vis differential spectra upon 530 nm LPL irradiation with the spectrum upon unpolarized light irradiation subtracted as a baseline. The orientation of the LPL light (i.e., the angle between the polarization of the light beam switching the sample and the polarization of the light beam of the spectrometer) is given in the legend on panel (b). d–i) Intensity changes of the absorption bands under LPL versus the angles between the light polarization of the UV–vis spectrometer and of the light for the sample irradiation. The absorption averaged in the range of 300–350 nm is shown in (d,g), indicating the intensity of the *trans* π–π* band; 390–410 nm in (e,h), indicating the intensity of the *cis* n–π* band and 440–450 nm in (f,i), indicating the intensity of the *trans* n–π* band. The sample is irradiated with violet light (405 nm) in (d–f), resulting in the *trans*‐rich state, and with green light (530 nm) in (g–i), resulting in the *cis*‐rich state. The black spheres are the experimental data, the green and violet lines are the fits with a sine curve.

In the next step, the orientation of the photochromic moiety as result of the polarization of the LPL irradiation is explored. In line with studies focusing on the photoalignment in liquid crystalline or polymeric films,^[^ ^50^, ^51^, ^52^, ^53^ ^]^ we use the data obtained from UV–vis spectroscopy to quantify the alignment. The polarized UV–vis spectra of the sample upon irradiation with 405 nm LPL with various orientation of the polarization are shown in **Figure** **3**a. The data show that the polarization of the light causing the azobenzene isomerization has a small but clear impact on the polarized UV–vis spectra. By irradiation with 405 nm, the *trans* isomers dominate. The transition dipole moments (TDMs) of the π–π* transition and of the n–π* transition of the fluorinated azobenzene molecule in the *trans* isomer are extended along the long axis of the azobenzene moiety (see Figure S2, Supporting Information, see also refs. [54, 55, 56] and see Figure 1b). As a result, under irradiation with 405 nm LPL (by exciting the n–π* transition) and a polarization angle of 0° (between the light exciting the sample and the spectrometer light), the azobenzene moieties preferentially orient perpendicular to the light polarization. This can be seen in the polarized UV–vis spectra, Figure 3d–f: The n–π* excitation (at 450 nm) and the π–π* excitation (at 320 nm) have decreased intensities for parallel polarization (0° and 180°) and increased intensities for perpendicular polarization (90° and 270°).

Upon exposure to polarized light of 530 nm (i.e., in the *cis*‐rich state), a similar effect can be observed. The dipole moment of the n–π* transition for the *cis* isomer is roughly oriented along the long axis of the molecules as well (see Figure S2, Supporting Information. Note, although the *cis* isomer is bent, the molecules possess a long axis connecting the two most distant atoms). This means, the molecules also orient under 530 nm LPL such that their long axis is perpendicular to the light polarization. This orientation can be visualized by polarized UV–vis spectroscopy, Figure 3g–i. In comparison to the spectral changes upon violet LPL, the changes upon green LPL are smaller.

From the polarized UV–vis spectra, the amount of the orientation can be estimated. We assume the system is only composed of molecules that are either parallel or perpendicular to the light polarization of the spectrometer. Specifically, the TDMs of the molecules should be either parallel or perpendicular to the light polarization. When the TDM is perpendicular to the light polarization, the molecules cannot be excited and do not absorb light. On the other hand, parallel orientation of the TDM of molecules permits the excitation and, thus, efficient absorption of the light. The absorbance change at 320 nm (Figure 3a) from 1.22 to 0.92 upon violet and green light irradiation is correlated with the *trans*‐yield of 89% and 19% upon violet and green light irradiation with randomly oriented TDM. For simplification, we assume that randomly oriented means 50% oriented parallel and 50% perpendicular to the light orientation. The perpendicularly oriented molecules do not contribute to the UV–vis spectra, since they do not absorb the light. Thus, an absorption unit (abs. u.) change of 0.01 at 320 nm in Figure 3a corresponds to a change of 2.3% of *trans*‐isomers parallel to the light polarization. Based on this estimation, the absorbance changes of 0.0186 abs.u. between the parallel and perpendicular polarization (Figure 3d) corresponds to an orientation change of the *trans* isomers by 4.3%. (Under the assumption that the *trans‐cis* ratio is not affected by the orientation of the polarization.) This means the light polarization causes the *trans* isomers to oriented preferably perpendicular to the polarization with a ratio of approximately 51.1%:48.9% (perpendicular:parallel), instead of 50:50. Please note, the orientation of the azo moieties can be reversibly adjusted.

**Figure 4:**
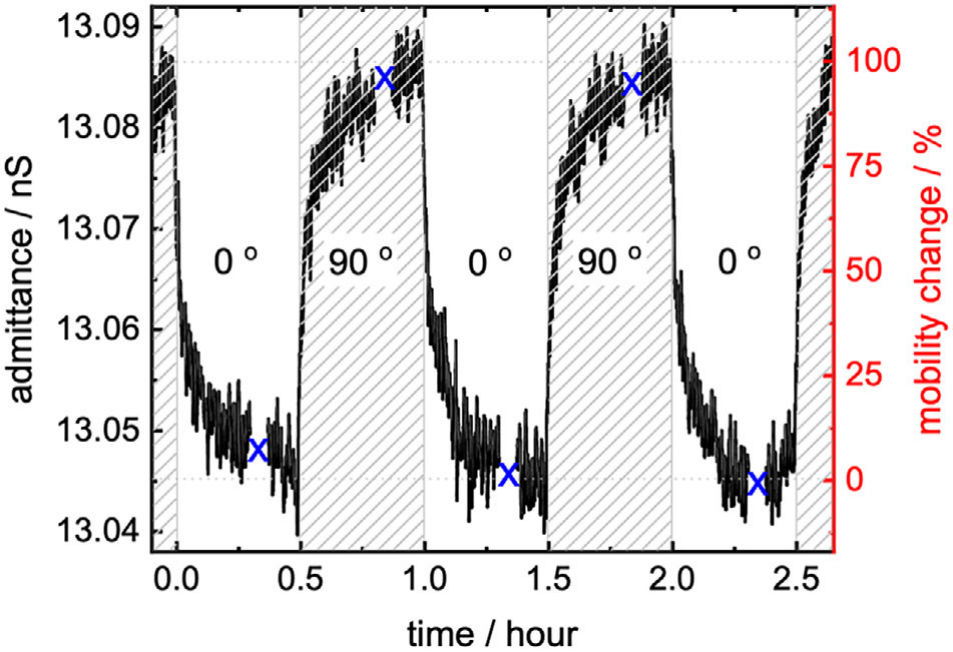
The ionic admittance versus time for different light irradiation angles, i.e., for LPL of 450 nm with the polarization parallel (0°) and perpendicular (90°) to the conduction direction. The frequency is 1 Hz. The blue crosses mark when the full impedance spectra (500 MHz to 0.5 Hz) were measured, see Figure S4 (Supporting Information). The scale on the right‐hand side shows the normalized changes of the ion mobility.

Taking advantage of the preferential orientation of the azobenzene side groups, we determine the mobility of the molecules in the pores and its polarization‐induced changes. To this end, we embedded molecular ions of type 1‐butyl‐3‐methylimidazolium bis(trifluoromethylsulfonyl)imide, i.e., [BMIM] cations and [TFSI] anions, in the pores. The charge transfer of the IL of type [BMIM][TFSI] is based on vehicle transport,^[^ ^53^ ^]^ where the (entire) cations and anions migrate through the medium and transport the charge of +1e and −1e. This mechanism arises because neither the cation nor the anion contains loosely bound, mobile ions, such as protons, that could facilitate charge transport by other means. Thus, the charge mobility corresponds to the molecular mobility. The loading was performed from solution, following previously optimized loading processes,^[^ ^57^, ^58^ ^]^ resulting in ≈5% [BMIM][TFSI]‐pore‐filling. The impedance spectroscopy data of the sample are shown in **Figure** **4** and Figures S4 and S5 (Supporting Information). By analyzing the Nyquist plot data with the appropriate equivalent circuit^[^ ^59^ ^]^ results in a resistance of 62.74 MΩ. For comparison, it was verified that the SURMOF sample without guest loading shows no conductivity, neither ionic nor electronic, Figure S6 (Supporting Information). Thus, the determined conductivity and mobility can be solely attributed to the cationic and anionic guests in the pores. With the determined film thickness of 350 nm and the electrode geometry, this corresponds to a conductivity of 269.47 nS m^−1^. From the pore fillings of 5%, the free MOF pore volume of 54.7% and a volume of the [BMIM][TFSI] ion pair of 4.84 × 10^−28^ m^3^, the average mobility of the molecular ions is determined to 1.49 × 10^−14^ m^2^ V^−1^ s^−1^.

The light polarization is switched between parallel and perpendicular to the direction of the electric field, which is the direction of the ionic conduction, Figure 4. The recorded data of the admittance (i.e., reciprocal of impedance, also referred to as complex conductance) show that the admittance of the ions in the SURMOF pores under 405 nm LPL irradiation is larger in the direction perpendicular to the light polarization than in the direction parallel to the light polarization. The difference between the two light irradiations is ≈0.3%. (This corresponds to a polarization‐induced admittance difference of ≈0.0365 nS, which is significantly larger than the standard deviation of the data of ≈0.002 nS, determined at the stable parts of the curve, i.e., in the 5 min range before the polarization change.) Since the ion mobility and the admittance are proportional for ionic conduction of aprotic ionic liquids (where the charge transport occurs only via vehicle transport),^[^ ^60^ ^]^ the mobility of the guest molecules in the MOF is also affected by 0.3%. This means the mobility of the ions in the pores is larger in the direction perpendicular to the light polarization, compared to the mobility parallel to the light polarization.

The change of the mobility can be understood by the view on the MOF pore along the ionic conduction direction, see Figure 1b bottom. For the MOF film grown in [001] direction perpendicular to the substrate surface (see Figure 1a) and the ion conduction parallel to the surface, the ion transport goes along the MOF pores in [100] direction, as shown in the bottom part of Figure 1b. In case the LPL polarization is parallel to the conduction direction, the azobenzene groups are oriented so that they hinder the motion in this direction. For the LPL polarization perpendicular to the conduction direction, the azobenzene groups are oriented such that they do not sterically hinder the molecular motion in this direction. Please note that in comparison to Figure 1, in which 100% of the all the molecules are oriented and also 100% of the molecules are *trans* configured, in our situation, the excess of the oriented molecules is only ≈4.3% and the *trans* azobenzene content is 89%.

Moreover, we like to stress that the MOF film is composed of many domains which are oriented with their crystalline [001] direction perpendicular to the surface (see Figure 1a), but these domains have no preferred crystalline orientation in the surface. A combination of the linear‐polarized light effects with films possessing an in‐plane orientation^[^ ^61^, ^62^ ^]^ may be beneficial in for future studies.

For gaining more insights on the molecular level, focusing on the IL mobility dependence on the anisotropic linker orientations in the Cu_2_(F_2_AzoBDC)_2_(dabco), molecular dynamics (MD) simulations on the atomistic level were performed. From the experimental reference (≈90% *trans* for azobenzene groups), we have adopted an all‐*trans* configuration when generating the MOF structure. The *trans‐*F_2_AzoBDC linker has a linear extension of ≈1 nm, which is too long to be perfectly aligned within one unit cell (with a size of 1.1 nm × 1.1 nm × 0.97 nm). Instead, the linkers with the azobenzene side groups are forced to protrude into the pores, where aromatic rings of the BDC backbones and of the azo side groups stack as azo‐BDC and azo‐azo complexes within a single pore as well as across neighboring pores. This is referred to as “natural configuration” for an empty MOF. Noteworthy, the azo groups already have a preferred orientation for the *x*‐ and *y*‐linkers. Force‐field modelling yields a 27° and 17° rotation for *x*‐ and *y*‐linkers, which agrees well with DFT‐optimized structures from ref. [43] (24° and 17°, Figure S9, Supporting Information).

**Figure 5:**
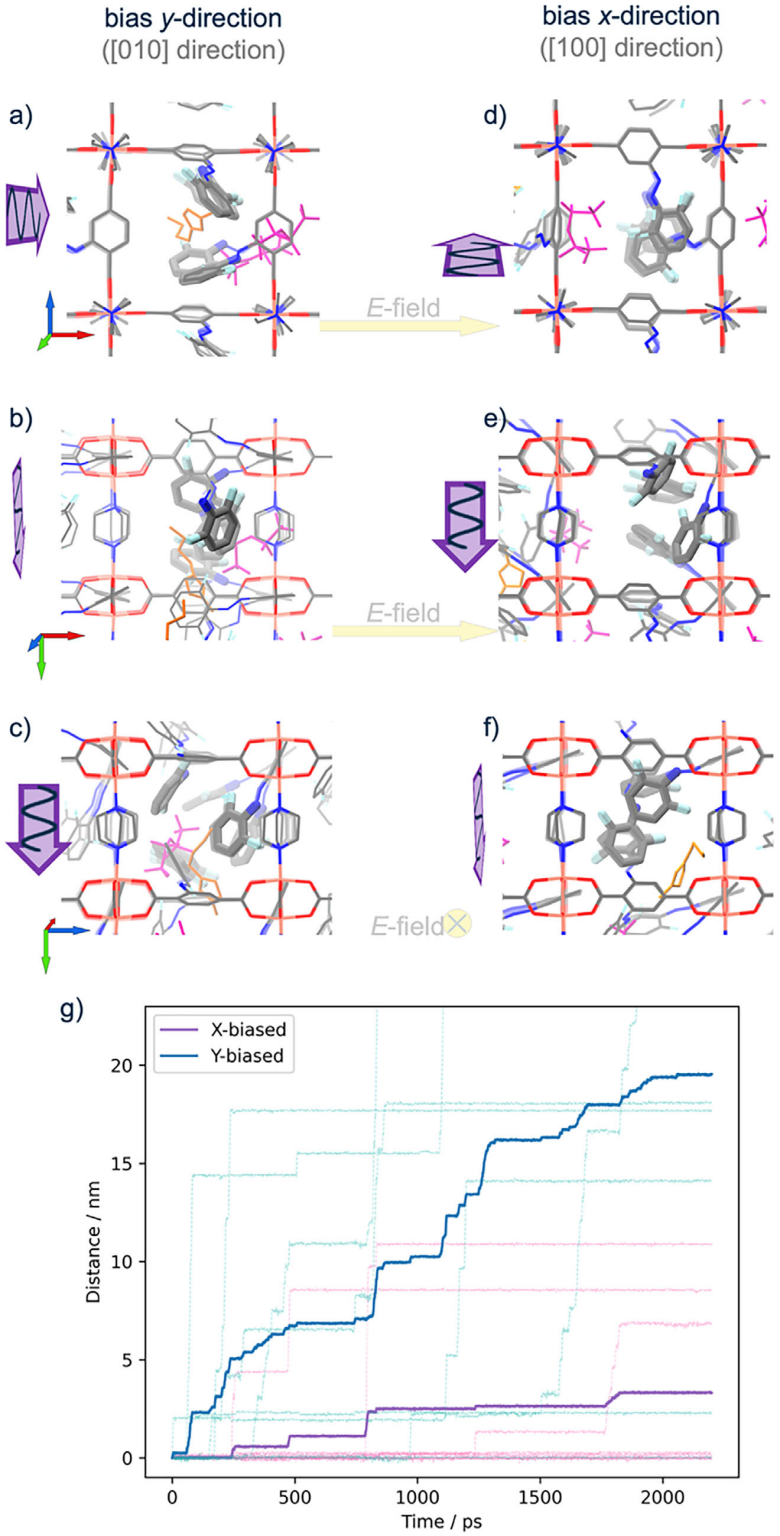
Simulated aligned MOF structure with the alignment bias along the *y*‐direction ([010] direction) shown in (a–c) and along the *x*‐direction ([100] direction) in (d–f). The alignment (see text and methods) mimics the photoalignment by LPL, which is indicated by the violet arrows, in analogy to Figure 1b. (This means, the light propagates in [001] direction where the light is polarized in [010] direction (left, (a–c)) and in [100] direction (right, (d–f)).) Spontaneous stacking of the linker moieties within MOF pores are accentuated by thickened azo groups. The view directions on the MOF structure are along [001] in (a,d), [010] direction in (b,e), and [100] direction in (c,f). See also tripods, which indicate the crystal directions like in Figure 1b; [100] ‐ red, [010] ‐ blue, and [001] – green. The IL transport, with [BMIM] in orange and [TFSI] in magenta, through the MOF is driven by an electric field in [100] direction, marked by the yellow arrows. g) The travelled distance of the ions as function of simulation time for the MOF aligned in different directions, see legend. The data from the individual trajectories (thin lines) and the average (thick lines) are shown. The travelled IL distance in the *y‐*biased MOF (blue; i.e., mimicking LPL with [010] polarization, that is perpendicular to the charge transport direction) is ≈6 times larger than the travelled IL distance in the *x‐*biased MOF (violet; i.e., mimicking LPL with [100] polarization, that is parallel to the charge transport direction). The entire plot is also shown in Figure S12 (Supporting Information).

To mimic the photoalignment, an artificial bias was applied to enforce the rotation of the F_2_AzoBDC linkers, respectively for the *x*‐linkers (with the backbone along the [100] direction) and for the *y*‐linkers (along the [010] direction). To this aim, a predominant artificial parabolic potential (40 kcal mol^−1^, centered at 90° versus the native dihedral of 1.2 kcal mol^−1^, centered at 0°) was applied, which rotates the C‐C‐C‐O dihedral angle in the BDC backbone of the F_2_AzoBDC, which rotates the entire linker, including the azobenzene side group. The aligned MOF structures are shown in **Figure** **5**. In a fully flexible relaxations of the MOF structure where the bias is only applied to either the *x*‐ or *y*‐linkers, the forced rotation of the linkers results in the aromatic ring in the BDC backbone essentially parallel to the [001] direction, perpendicular to the Cu‐BDC‐planes. In detail, the linker aligns with an angle of smaller than 7°. For the other non‐biased half of the linkers, anisotropic packing configurations for the azobenzene side groups emerges through π–π stacking, see Figure 5 and Figure S8 (Supporting Information).

For investigating the ion motions under an external electric field (*E*‐field), the positions of the F_2_AzoBDC linkers and Cu‐paddlewheels were fixed (i.e., the alignment was frozen), and the other parts of the MOF (dabco pillar linkers) were flexible. Across the framework, the ILs were driven by an electric field in [100] direction. Compared to the “natural configuration”, *y‐*linkers under bias rotate away from the pore center, to allow a boosted mobility of guest molecules (see Figure 5c). In contract, the *x‐*bias causes the *x‐*linkers across the MOF pores to stack with other moieties, blocking the IL flow (see Figure 5f). Detailed discussion of the MD simulation is included in the Experimental Section and in the Supporting Information. As main finding, the simulations show that the IL mobility (in [100] direction) in the MOF with the alignment in [010] direction (i.e., in *y*‐bias) is ≈6 times higher than in the MOF with the alignment in [100] direction (i.e., in *x*‐bias). This is in agreement with the experiment, considering also the differences in the alignment and isomerization percentages. It should be noted that a similar result was also found with fully flexible MD simulations (Figures S10 and S11, Supporting Information).

In the experiments, the small loading of IL of 5% was chosen to avoid effects like mutual pore blockage and conduction collapse, which may happen at large pore loadings in MOFs.^[^ ^58^, ^63^, ^64^ ^]^ To allow more general statements, MD simulations with charged small spherical particles were also performed, Figure S13 (Supporting Information). These simulations for rather small ions show similar alignment differences as for the fully atomistic simulations for large ions of [BMIM][TFSI]. This indicates that the observed (photo‐)alignment effect is not limited to the studied IL.

Apart from the photoalignment in thin films on solid substrates, the photoalignment (as property of the material under irradiation with light) can also occur in other forms the material, like free‐standing films or single crystals. Please note that the light‐penetration depth (which is typically in the range of 1µm or less)^[^ ^65^, ^66^ ^]^ does not permit the homogeneous illumination, photoswitching and, thus, photoalignment of large single crystals, pellets, or thick films.

In the present proof of principle study, we introduce the light polarity as a further parameter to control the material properties—in addition to the light intensity and color. While the effect is demonstrated, we believe the magnitude, this means the anisotropy ratio and the ON–OFF difference, needs to be enhanced before targeting real applications. In addition to the information transfer and encryption, where the light polarization can be used as an additional input, potential visionary applications include smart membrane separation and smart electrochemical devices such as photo‐switchable batteries, fuel cells, or supercapacitors, where the ion transport is modulated by light. We believe the present work, which introduces the photoalignment in porous materials to induce anisotropic and directional mobilities in the pores will inspire future work with (hopefully) larger anisotropy ratios of the mobility, allowing various applications.

## 3 Conclusion

The directional control of the mobility of guest molecules inside the nanopores of a porous material is presented by using linearly polarized light. The material is a metal–organic framework thin film with photoresponsive fluorinated azobenzene side groups connected to the framework. By exciting the n–π* transition of the azobenzene moieties with LPL, the molecules dynamically reorient until they do adopt an orientation in which their transition dipole moments are perpendicular to the light polarization and thus the azobenzenes cease to absorb the LPL. This induces an anisotropy of the initially isotropic MOF structure and the pore channels. This anisotropy of the pore channels causes a directionality of the motion of guest molecules within the pores. As a result, we could demonstrate that for the molecules within the pores, the mobility in the direction parallel to the polarization of the light is smaller than perpendicular to the polarization of the light. Changing the light polarization consequently alters the direction of the mobility as well.

For the first time, our study shows that in addition to wavelength and intensity, the polarization of light can be employed as a decisive parameter to control the properties of a photoresponsive nanoporous material. We foresee that in addition to MOF films, this can be applied to other crystalline or amorphous photoresponsive materials with various functional components. In addition to the anisotropic mobility, further properties such as proton conduction, macroscopic diffusion, and other properties can be directed by linearly polarized light, opening promising avenues for designing more efficient energy conversion as well as separation materials.

## 4 Experimental Section

### Chemicals and Substrates

Copper acetate monohydrate (99.9%), 11‐mercapto‐l‐undecanol (97%), and 1,4‐diazabicyclo[2.2.2]octane (98%) were purchased from Alfa Aesar, ionic liquid 1‐butyl‐3‐methylimidazolium bis(trifluoromethylsulfonyl)imide ([BMIM][TFSI]) from Sigma–Aldrich, ethanol (EtOH, 99.8%) from VWR, copper nitrate trihydrate (99.9%), 99.8%) from VWR and N,N‐dimethylformamide (DMF) was obtained from Merck. The photoswitchable MOF linker 2‐((2,6‐difluorophenyl)diazenyl)terephthalic acid (F_2_AzoBDC) was prepared as described before.^[^ ^30^ ^]^

The substrates are glass substrates with deposited interdigitated gold electrodes (IDEs, where the electrodes are parallel to each other) for EIS, XRD, and SEM measurements from Metrohm. The gap width is 10 micrometer and the total gap length 1.69m. These substrates were cleaned and functionalized using a UV‐ozone cleaner to support the SURMOF growth. Quartz glass and gold‐coated silicon wafer (from PVD‐Beschichtungen, Silz, Germany) were used for the UV–vis and IR spectroscopy. The gold‐coated silicon wafers were functionalized with an OH‐terminated self‐assembled monolayer by immersing the clean gold surfaces into ethanolic solutions of 11‐mercapto‐l‐undecanol for 24 h.

### Synthesis of SURMOF and IL Loading

The Cu_2_(F_2_AzoBDC)_2_(dabco) SURMOF films were prepared on different substrates in a layer‐by‐layer (LBL) method.^[^ ^67^ ^]^ The ethanolic solutions contained 1 mm copper acetate as metal source and a mixture solution of 0.2 mm F_2_AzoBDC and 0.2 mm dabco as linker solution. The substrates were kept in the metal solution for 15 min and in the organic linker solution for 25 min. The washing with ethanol in between was performed for 2 min each. All samples on gold coated silicon wafers (for IRRAS) were prepared in 40 synthesis cycles. The samples on quartz (for UV–vis) were prepared in 20 synthesis cycles, and the samples on IDE substrates (for EIS) were prepared in 50 synthesis cycles. The numbers of synthesis cycles, and thus the thicknesses, are optimized for the respective techniques.

The [BMIM][TFSI] loading of the SURMOFs was performed following a previously optimized method.^[^ ^57^ ^]^ The SURMOF samples were loaded from a 5% [BMIM][TFSI] solution in EtOH for 20 h. Then, the sample surface was washed to remove residual excess [BMIM][TFSI] on the surface by dipping in a fresh ethanol solution for 2 s. Then, the samples were dried in a flow of pure nitrogen, removing (volatile) ethanol from the pores.

### Synthesis of Cu_2_(F_2_AzoBDC)_2_(dabco) Powder MOF

The Cu_2_(F_2_AzoBDC)_2_(dabco) MOF powder was prepared using the solvothermal method.^[^ ^43^ ^]^ Copper(II) nitrate trihydrate (86.9 mg, 0.36 mmol), F_2_AzoBDC (110.35 mg, 0.36 mmol), and dabco (20.2 mg, 0.18 mmol) were dissolved in a mixture of N,N‐dimethylformamide (DMF) and ethanol (1:1 v/v, 50 mL total). The solution was sonicated for 10 min and then transferred into a 100 mL Teflon lined stainless steel autoclave and heated at 100 °C for 48 h. After cooling to room temperature, the product was washed with DMF and EtOH twice and dried under a flow of dry nitrogen.

### Characterization

X‐ray diffraction (XRD) analyses were performed with a Bruker D8 Advance diffractometer (for out‐of‐plane XRD) and a Bruker D8 Discover (for in‐plane XRD) diffractometer. In both cases, the X‐ray wavelength was λ = 0.154 nm (Cu‐Kα). For the out‐of‐plane XRD and powder, the diffractograms were recorded in the range of 3–20° with a step size of 0.01° and an accumulation time of 4 s per step. The in‐plane XRD data were measured using a *Bruker D8 Discover* diffractometer, equipped additionally with a tilt‐stage, a quarter Eulerian cradle and 2.3° Sollar‐slits. The range was 3–20° with a step size of 0.02°.

The surface and cross‐sectional scanning electron microscope (SEM) images were recorded on/with TESCAN VEGA3.

Infrared reflection‐absorption spectroscopy (IRRAS) was performed with a Bruker VERTEX 80 device in grazing incidence reflection mode at an angle of incidence of 80° relative to the surface normal.

Angle‐resolved polarized UV–vis spectra were recorded with a Cary5000 spectrometer with a UMA unit from Agilent. In the experiment, the incident polarization was fixed to be vertical by using a linear polarizer in the beam path of the UV–vis spectrometer. The spectral resolution was set to 1 nm, the accumulation time was 1 s.

For irradiating the sample, light of 405 and 530 nm was polarized with a linear polarizer, and the polarizer was placed on a rotation stage and clockwise rotated by 360° with a step size of 30°. Each irradiation time was 30 min. The parameters and light intensities of the used LEDs are 24.6 µW/mm^2^ (405 nm, M405LPl, Thorlabs) and 9.46 µW mm^−2^ (530 nm, M530L4, Thorlabs), respectively. The linear polarizers were also purchased from Thorlabs. (WP12L‐UB was used for the UV–vis‐spectroscopy LPVISEIOO‐A combined with a computer‐controlled rotation unit (ELL14K) for the LPL impedance spectroscopy experiments.)

The ionic conduction and the ion mobility were assessed by electrochemical impedance spectroscopy with a Zurich Instruments MFIA Impedance Analyzer. The sample on the IDE‐ substrate was in a home‐built cell^[^ ^43^, ^68^ ^]^ which was purged with pure nitrogen at room temperature. The sample was irradiated in situ through a quartz window. The automatic angle controller and polarizer, see above, were used. The impedance spectra were recorded in a range from 500 kHz to 0.5 Hz with a peak‐to‐peak amplitude of 300 mV. The impedance versus time data were recorded at a constant frequency of 1 Hz and a current range of 1 µA.

### Transition Dipole Moment Calculations

The quantum mechanical calculations of the directionality of the TDM were performed using the time‐dependent density functional theory calculations (TD‐DFT). For that B3LYP functional^[^ ^69^ ^]^ with def2‐TZVP basis set,^[^ ^70^ ^]^ as implemented in TURBOMOLE (version 7.3)^[^ ^71^ ^]^ were utilized. Global minima structures of *trans* and *cis* isomers of F_2_AzoBDC were considered. The character of optical transitions was estimated using the transition molecular orbitals (see Table S1, Supporting Information).

### Molecular Dynamics Simulations of IL Transport with Biased Rotation of Azobenzene Side Groups

All‐atom models of the Cu_2_(F_2_AzoBDC)_2_(dabco) SURMOF as a 4x4x4 supercell as well as 8 pairs (5% occupancy of free pore volume) of [BMIM][TFSI] were merged by the software package Packmol.^[^ ^72^ ^]^ The SURMOF was parameterized in UFF4MOF forcefield by lammps‐interface^[^ ^73^ ^]^ based on an experimental CIF structure of the Cu_2_(F_2_AzoBDC)_2_(dabco). The IL force field parameters were based on DFT data, which were compatible with the OPLS‐aa and UFF4MOF in our previous investigations.^[^ ^58^, ^74^, ^75^ ^]^ The relaxation of the MOF structure before and after the application of specific biases corresponded to 2 ns, and linker alignment was extracted by the C(x/y)‐C(Azo) vector around the azo bond. Due to the small pore windows of the SURMOF and large cross‐sectional areas of the cations and anions in the IL, frequent occurrences of kinetic trapping significantly hindered data collection for the IL transport. To counter this, an artificial shrinkage of the cations and anions to 80% was incorporated into the model by reducing the atom‐wise van der Waals radii and bond lengths. In semi‐rigid MOF simulations, the dabco pillar linker was allowed to rotate to practically prevent kinetic trapping when simulating the IL transport.

As individual ions drift across MOF pores driven by an external electric field with a strength of 12 V/nm, the kinetics of individual IL molecules were averaged and fitted linearly as a steady ion flow was established. The conductivity was determined as Λ = *F*Σ_*i*_(*q_i_* 〈*v_i_* 〉) by summing the contribution from both cations and anions. Here in the equation, *F* is the Faraday constant, *q_i_* s correspond to the net charge of [BMIM] and [TFSI] and 〈*v_i_* 〉s are mobility taken average from time windows of 1.5 ns trajectory in a (1 ns relaxation + 5 ns production) MD simulation.

For reference, all‐flexible simulations are also performed with a 1 ns timespan. There, due to the intense interaction of IL impacting against the *x*‐ and *y‐*linkers, the linker alignment show a broad distribution, potentially overshadowing the effect from photoalignment, in Figure S10 (Supporting Information). As a result, we discuss the IL mobilities based on (semi‐)rigid MOF simulations. Albeit the ILs’ impacting the linkers considerably alters the linker alignment, particularly in the case of all‐flexible simulation (7° in rigid vs 32° in flexible), a sequentially reducing *y‐* vs *x‐*biased IL mobility of 3.7:1 (40 kcal mol^−1^), 3:1 (10 kcal mol^−1^), 1.2:1 (4 kcal mol^−1^) extrapolates well from the rigid simulation, in Table S2 (Supporting Information).

An additional note from all‐flexible simulation is that the forced *trans‐cis* transition is observed. By tracing C(x/y)‐N‐N‐C(Azo) dihedral angle as a time series, *trans*‐*cis* switching showed identical behavior under *x*‐ and *y*‐biases, where the *trans* conformation always dominated with >90% population. This was also consistent with the mostly *trans* (89%) configuration from the experiment.

## Conflict of Interest

The authors declare no conflict of interest.

## Supporting information



Supporting Information

Supporting Information

## Data Availability

The data that support the findings of this study are available on request from the corresponding author. The data are not publicly available due to privacy or ethical restrictions.

## References

[1] C. W. Mullineaux , Photochem. Photobiol. 2008, 84, 1310. 18764904 10.1111/j.1751-1097.2008.00420.x

[2] C. J. Stefan , W. S. Trimble , S. Grinstein , G. Drin , K. Reinisch , P. De Camilli , S. Cohen , A. M. Valm , J. Lippincott‐Schwartz , T. P. Levine , D. B. Iaea , F. R. Maxfield , C. E. Futter , E. R. Eden , D. Judith , A. R. van Vliet , P. Agostinis , S. A. Tooze , A. Sugiura , H. M. McBride , BMC Biol. 2017, 15, 102. 29089042 10.1186/s12915-017-0432-0 PMC5663033

[3] C. Echevería , K. Tucci , R. Kapral , J. Phys.: Condens. Matter 2007, 19, 065146.

[4] L. Ebersberger , T. Schindler , S. A. Kirsch , K. Pluhackova , A. Schambony , T. Seydel , R. A. Böckmann , T. Unruh , Front. Cell Develop. Biol. 2020, 8, 579388. 10.3389/fcell.2020.579388 PMC7649217 33195218

[5] Y. Senju , S. Suetsugu , Membranes 2023, 13, 904. 38132908 10.3390/membranes13120904 PMC10744542

[6] B. L. Feringa , W. R. Browne , Molecular Switches, Wiley, Hoboken, NJ 2011.

[7] Z. L. Pianowski , Molecular Photoswitches: Chemistry, Properties, and Applications, vol 2, Wiley, Hoboken, NJ 2022.

[8] M.‐M. Russew , S. Hecht , Adv. Mater. 2010, 22, 3348. 20422653 10.1002/adma.200904102

[9] J. Morstein , A. C. Impastato , D. Trauner , ChemBioChem 2021, 22, 73. 32790211 10.1002/cbic.202000449

[10] Z. Wang , A. Knebel , S. Grosjean , D. Wagner , S. Bräse , C. Wöll , J. Caro , L. Heinke , Nat. Commun. 2016, 7, 13872. 27996002 10.1038/ncomms13872 PMC5187437

[11] G. Scandura , S. Eid , A. A. Alnajjar , T. Paul , G. N. Karanikolos , D. Shetty , K. Omer , R. Alqerem , A. Juma , H. Wang , H. A. Arafat , L. F. Dumée , Mater. Adv. 2023, 4, 1258.

[12] F. Marlow , K. Hoffmann , J. Caro , Adv. Mater. 1997, 9, 567.

[13] K. Müller , A. Knebel , F. Zhao , D. Bléger , J. Caro , L. Heinke , Chem. – Eur. J. 2017, 23, 5434. 28370503 10.1002/chem.201700989

[14] Y. Jiang , W. Danowski , B. L. Feringa , L. Heinke , Angew. Chem., Int. Ed. 2023, 62, 202214202. 10.1002/anie.202214202 PMC10107543 36367076

[15] J. A. M. Lugger , P. P. Marín San Román , C. C. E. Kroonen , R. P. Sijbesma , ACS Appl. Mater. Interfaces 2021, 13, 4385. 33430592 10.1021/acsami.0c19180 PMC7844832

[16] L. L. Gong , W. T. Yao , Z. Q. Liu , A. M. Zheng , J. Q. Li , X. F. Feng , L. F. Ma , C. S. Yan , M. B. Luo , F. Luo , J. Mater. Chem. A 2017, 5, 7961.

[17] K. Müller , J. Helfferich , F. L. Zhao , R. Verma , A. B. Kanj , V. Meded , D. Bléger , W. Wenzel , L. Heinke , Adv. Mater. 2018, 30, 1706551 10.1002/adma.201706551 29315923

[18] A. B. Kanj , A. Chandresh , A. Gerwien , S. Grosjean , S. Bräse , Y. Wang , H. Dube , L. Heinke , Chem. Sci. 2020, 11, 1404.

[19] H.‐Q. Liang , Y. Guo , Y. Shi , X. Peng , B. Liang , B. Chen , Angew. Chem. Int. Ed. 2020, 59, 7732, 10.1002/anie.202002389. 32090427

[20] W. Szymański , J. M. Beierle , H. A. V. Kistemaker , W. A. Velema , B. L. Feringa , Chem. Rev. 2013, 113, 6114. 23614556 10.1021/cr300179f

[21] A. Kerckhoffs , M. J. Langton , Chem. Sci. 2020, 11, 6325. 32953027 10.1039/d0sc02745f PMC7472928

[22] G. S. Hartley , R. J. W. Le Fèvre , J. Chem. Soc. 1939, 119, 531.

[23] H. M. D. Bandara , S. C. Burdette , Chem. Soc. Rev. 2012, 41, 1809. 22008710 10.1039/c1cs15179g

[24] C. Knie , M. Utecht , F. Zhao , H. Kulla , S. Kovalenko , A. M. Brouwer , P. Saalfrank , S. Hecht , D. Bléger , Chemistry 2014, 20, 16492. 25352421 10.1002/chem.201404649

[25] D. Bleger , J. Schwarz , A. M. Brouwer , S. Hecht , J. Am. Chem. Soc. 2012, 134, 20597. 23236950 10.1021/ja310323y

[26] K. Kumar , C. Knie , D. Bléger , M. A. Peletier , H. Friedrich , S. Hecht , D. J. Broer , M. G. Debije , A. P. H. J. Schenning , Nat. Commun. 2016, 7, 11975. 27375235 10.1038/ncomms11975 PMC4932179

[27] O. S. Bushuyev , A. Tomberg , T. Friščić , C. J. Barrett , J. Am. Chem. Soc. 2013, 135, 12556. 23924402 10.1021/ja4063019

[28] D. Hermann , H. A. Schwartz , M. Werker , D. Schaniel , U. Ruschewitz , Chem ‐ Eur. J. 2019, 25, 3606. 30633421 10.1002/chem.201805391

[29] B. R. Donovan , V. M. Matavulj , S.‐k. Ahn , T. Guin , T. J. White , Adv. Mater. 2019, 31, 1805750. 10.1002/adma.201805750 30417450

[30] S. Castellanos , A. Goulet‐Hanssens , F. Zhao , A. Dikhtiarenko , A. Pustovarenko , S. Hecht , J. Gascon , F. Kapteijn , D. Bleger , Chem ‐ Eur. J. 2016, 22, 746. 26617393 10.1002/chem.201503503

[31] H. Furukawa , K. E. Cordova , M. O'Keeffe , O. M. Yaghi , Science 2013, 341, 1230444. 23990564 10.1126/science.1230444

[32] S. Kaskel , The Chemistry of Metal‐Organic Frameworks: Synthesis, Characterization, and Applications, Wiley, Hoboken, NJ 2016.

[33] A. Modrow , D. Zargarani , R. Herges , N. Stock , Dalton Trans. 2012, 41, 8690. 22692132 10.1039/c2dt30672g

[34] L. Heinke , M. Cakici , M. Dommaschk , S. Grosjean , R. Herges , S. Bräse , C. Wöll , ACS Nano 2014, 8, 1463. 24400960 10.1021/nn405469g

[35] A. Knebel , L. Sundermann , A. Mohmeyer , I. Strauß , S. Friebe , P. Behrens , J. Caro , Chem. Mater. 2017, 29, 3111.

[36] S. Garg , H. Schwartz , M. Kozlowska , A. B. Kanj , K. Müller , W. Wenzel , U. Ruschewitz , L. Heinke , Angew. Chem., Int. Ed. 2019, 58, 1193. 10.1002/anie.201811458 30421842

[37] E. A. Dolgopolova , V. A. Galitskiy , C. R. Martin , H. N. Gregory , B. J. Yarbrough , A. M. Rice , A. A. Berseneva , O. A. Ejegbavwo , K. S. Stephenson , P. Kittikhunnatham , S. G. Karakalos , M. D. Smith , A. B. Greytak , S. Garashchuk , N. B. Shustova , J. Am. Chem. Soc. 2019, 141, 5350. 30840822 10.1021/jacs.8b13853

[38] T. Ikeda , J. Mater. Chem. 2003, 13, 2037.

[39] T. Akitsu , C. Ishioka , T. Itoh , Cent. Eur. J. Chem. 2009, 7, 690.

[40] T. D. Ebralidze , N. A. Ebralidze , M. A. Bazadze , Appl. Opt. 2002, 41, 78. 11900450 10.1364/ao.41.000078

[41] J. Eggert , Ber. Bunsenges. Phys. Chem. 1971, 75, 725.

[42] T. Koehler , I. Strauss , A. Mundstock , J. Caro , F. Marlow , J. Phys. Chem. Lett. 2021, 12, 8903. 34498886 10.1021/acs.jpclett.1c02489 PMC8450931

[43] A. B. Kanj , J. Bürck , N. Vankova , C. Li , D. Mutruc , A. Chandresh , S. Hecht , T. Heine , L. Heinke , J. Am. Chem. Soc. 2021, 143, 7059. 33915047 10.1021/jacs.1c01693

[44] O. Shekhah , H. Wang , S. Kowarik , F. Schreiber , M. Paulus , M. Tolan , C. Sternemann , F. Evers , D. Zacher , R. A. Fischer , C. Wöll , J. Am. Chem. Soc. 2007, 129, 15118. 18020338 10.1021/ja076210u

[45] L. Heinke , C. Wöll , Adv. Mater. 2019, 31, 1806324. 10.1002/adma.201806324 30701602

[46] Z. Zhang , K. Müller , S. Heidrich , M. Koenig , T. Hashem , T. Schlöder , D. Bléger , W. Wenzel , L. Heinke , J. Phys. Chem. Lett. 2019, 10, 6626. 31596091 10.1021/acs.jpclett.9b02614

[47] P. Qin , S. Okur , C. Li , A. Chandresh , D. Mutruc , S. Hecht , L. Heinke , Chem. Sci. 2021, 12, 15700. 35003601 10.1039/d1sc05249g PMC8654041

[48] Y. Li , A. Chandresh , H. H. Lin , N. Vankova , D. Mutruc , T. Heine , S. Hecht , L. Heinke , Adv. Mater. 2025, 37, 2419195. 40190219 10.1002/adma.202419195 PMC12232227

[49] Z. J. Zhang , D. H. Chen , D. Mutruc , S. Hecht , L. Heinke , Chem. Commun. 2022, 58, 13963. 10.1039/d2cc03862e 36453243

[50] N. Kawatsuki , H. Matsushita , T. Washio , J. Kozuki , M. Kondo , T. Sasaki , H. Ono , Macromolecules 2014, 47, 324.

[51] Y. Kawashima , M. Nakagawa , K. Ichimura , T. Seki , J. Mater. Chem. 2004, 14, 328.

[52] Y. Zakrevskyy , J. Stumpe , B. Smarsly , C. F. J. Faul , Phys. Rev. E 2007, 75, 031703. 10.1103/PhysRevE.75.031703 17500707

[53] T. Ube , H. Tsunoda , K. Kawasaki , T. Ikeda , Adv. Opt. Mater. 2021, 9, 2100053.

[54] T. Moldt , D. Brete , D. Przyrembel , S. Das , J. R. Goldman , P. K. Kundu , C. Gahl , R. Klajn , M. Weinelt , Langmuir 2015, 31, 1048. 25544061 10.1021/la504291n

[55] T. S. Yankova , N. A. Chumakova , D. A. Pomogailo , A. K. Vorobiev , Liq. Cryst. 2013, 40, 1135.

[56] A. Kunz , N. Oberhof , F. Scherz , L. Martins , A. Dreuw , H. A. Wegner , Chemistry 2022, 28, 202200972. 10.1002/chem.202200972 PMC9401047 35499252

[57] Z. Zhang , C. Li , A. Chandresh , L. Heinke , Ionics 2022, 28, 487.

[58] A. B. Kanj , R. Verma , M. Liu , J. Helfferich , W. Wenzel , L. Heinke , Nano Lett. 2019, 19, 2114. 30830791 10.1021/acs.nanolett.8b04694

[59] A. Chandresh , Z. Zhang , L. Heinke , Materials 2021, 14, 4352. 34442873 10.3390/ma14164352 PMC8399861

[60] MacFarlane, D. R. ; Kar, M. ; Pringle, J. M. , Fundamentals of Ionic Liquids: From Chemistry to Applications, Wiley, Hoboken, NJ 2017.

[61] P. Falcaro , K. Okada , T. Hara , K. Ikigaki , Y. Tokudome , A. W. Thornton , A. J. Hill , T. Williams , C. Doonan , M. Takahashi , Nat. Mater. 2017, 16, 342. 27918565 10.1038/nmat4815

[62] P. Thissen , J. Wohlgemuth , P. Weidler , D. Smilgies , L. Heinke , N. Schewe , M. Koenig , P. Krolla , C. Wöll , Adv. Funct. Mater. 2024, 34, 2301535.

[63] Z. J. Zhang , M. D. Liu , C. Li , W. Wenzel , L. Heinke , Small 2022, 18, 2200602, 10.1002/smll.202200602. 36002338

[64] M. Vazquez , M. Liu , Z. Zhang , A. Chandresh , A. B. Kanj , W. Wenzel , L. Heinke , ACS Appl. Mater. Interfaces 2021, 13, 21166. 33905243 10.1021/acsami.1c00366

[65] R. Haldar , L. Heinke , C. Wöll , Adv. Mater. 2020, 32, 1905227. 10.1002/adma.201905227 31763731

[66] Y. Z. Jiang , L. Heinke , Langmuir 2021, 37, 2. 33347762 10.1021/acs.langmuir.0c02859

[67] Z.‐G. Gu , A. Pfriem , S. Hamsch , H. Breitwieser , J. Wohlgemuth , L. Heinke , H. Gliemann , C. Wöll , Microporous Mesoporous Mater. 2015, 211, 82.

[68] C. Li , H. Schopmans , L. Langer , S. Marschner , A. Chandresh , J. Bürck , Y. Tsuchiya , A. Chihaya , W. Wenzel , S. Bräse , M. Kozlowska , L. Heinke , Angew. Chem., Int. Ed. 2023, 62, 202217377. 10.1002/anie.202217377 36515401

[69] A. D. Becke , J. Chem. Phys. 1993, 98, 5648.

[70] A. Schäfer , H. Horn , R. Ahlrichs , J. Chem. Phys. 1992, 97, 2571.

[71] S. G. Balasubramani , G. P. Chen , S. Coriani , M. Diedenhofen , M. S. Frank , Y. J. Franzke , F. Furche , R. Grotjahn , M. E. Harding , C. Hattig , A. Hellweg , B. Helmich‐Paris , C. Holzer , U. Huniar , M. Kaupp , A. Marefat Khah , S. Karbalaei Khani , T. Muller , F. Mack , B. D. Nguyen , S. M. Parker , E. Perlt , D. Rappoport , K. Reiter , S. Roy , M. Ruckert , G. Schmitz , M. Sierka , E. Tapavicza , D. P. Tew , et al., J. Chem. Phys. 2020, 152, 184107, 10.1063/5.0004635. 32414256 PMC7228783

[72] L. Martínez , R. Andrade , E. G. Birgin , J. M. Martínez , J. Comput. Chem. 2009, 30, 2157. 19229944 10.1002/jcc.21224

[73] P. G. Boyd , S. M. Moosavi , M. Witman , B. Smit , J. Phys. Chem. Lett. 2017, 8, 357. 28008758 10.1021/acs.jpclett.6b02532 PMC5253710

[74] Z. J. Li , Y. L. Xiao , W. J. Xue , Q. Y. Yang , C. L. Zhong , J. Phys. Chem. C 2015, 119, 3674.

[75] D. E. Coupry , M. A. Addicoat , T. Heine , J. Chem. Theory Comput. 2016, 12, 5215. 27580382 10.1021/acs.jctc.6b00664

