# Supplementary material for: Directing the Mobility of Guest Molecules in Nanoporous Materials by Linearly Polarized Light
Source: Adv Sci (Weinh). 2025 Nov 27;13(8):e03500. doi: 10.1002/advs.202503500 (PMC12884782; doi:10.1002/advs.202503500)
Supplement: Supplementary file 1 — Supporting Information [file ADVS-13-e03500-s002.docx]

Supporting Information

Directing the mobility of guest molecules in nanoporous materials by linearly polarized light

*Taher Al Najjar,^#^ Chun Li, ^#^ Yunzhe Jiang, ^#^ Anna Mauri, Modan Liu, Abhinav Chandresh, Anemar Bruno Kanj, Dragos Mutruc, Wolfgang Wenzel, Stefan Hecht, Mariana Kozlowska and Lars Heinke**

**
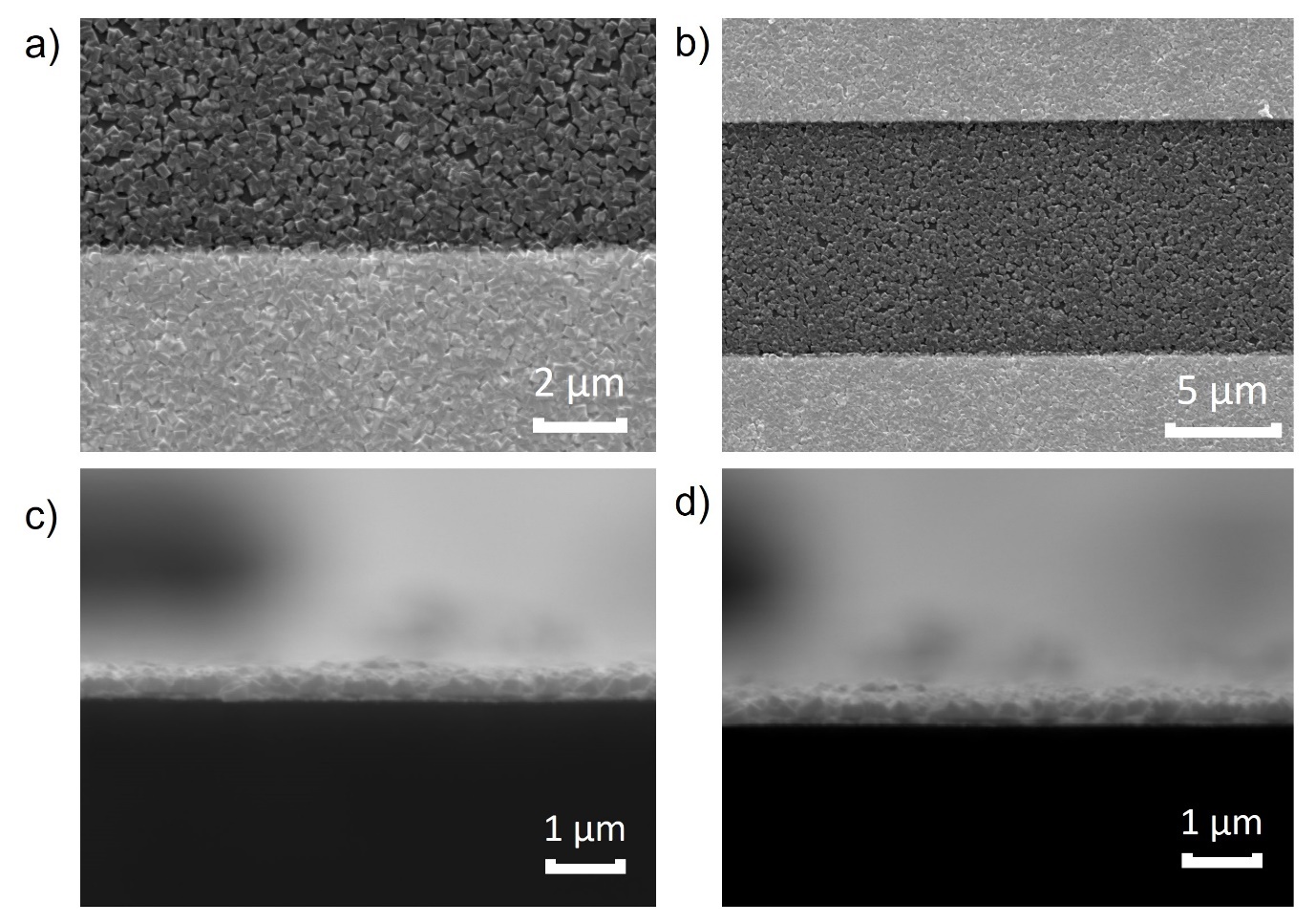
Figure S1.** Wide view SEM images of the sample in Figure 4. The gold electrodes are visible as bright stripes in **a)** and **b)**; side view of the broken sample is shown in **c)** and **d)**.

**
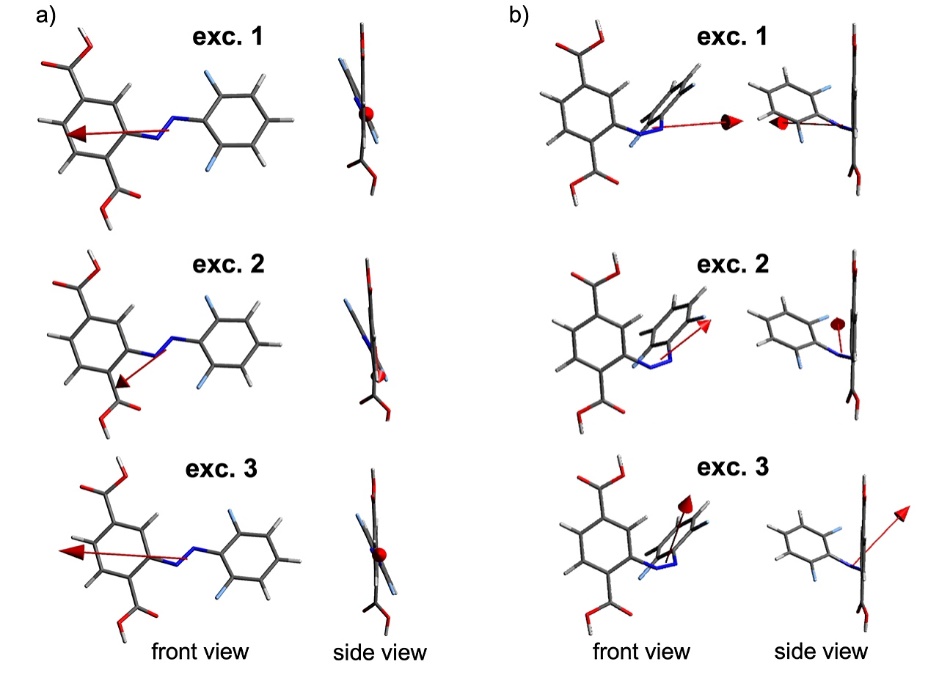
**

**Figure S2. TDM:** Transition dipole moment for the first three excitations of **a)** *trans* and **b)** *cis* isomer of the linker molecule. The red arrows indicate the TDM.

**Table S1**: Summary of electronic excitations (exc) for the *cis* and *trans* isomers considering the excitation wavelength, oscillator strength, orbital contribution and type of transition.

| *cis* | Wavelength (nm) | Oscillator strength | Main contribution | Type of transition |
| --- | --- | --- | --- | --- |
| exc1  exc2  exc3 | 467.484  370.244  312.894 | 0.03585  0.01449  0.00488 | HOMO → LUMO (77%)  HOMO → LUMO+1 (77%)  HOMO-1 → LUMO (95%) | nπ*  nπ*  ππ* |
| *trans* | Wavelength (nm) | Oscillator strength | Main contribution | Type of transition |
| exc1  exc2  exc3 | 519.983  346.589  327.255 | 0.00239  0.01471  0.58989 | HOMO → LUMO (94%)  HOMO → LUMO+1 (92%)  HOMO-1 → LUMO (87%) | nπ*  nπ*  ππ* |

The lowest-lying excitation has n−π* character for both isomers, with transitions at 520 nm (*trans*) and 467 nm (*cis*). This transition is symmetry-forbidden in the *trans* isomer, resulting in a low oscillator strength. The second excitation involves n-to-terephthalate ligand transitions, occurring at 347 nm (*trans*) and 370 nm (*cis*). The third excitation corresponds to a HOMO−1 to LUMO transition, with wavelengths of 327 nm (*trans*) and 313 nm (*cis*). Significant differences in oscillator strengths (0.590 for the *trans* isomer vs 0.005 for the *cis* isomer) arise due to the localization of the HOMO−1 orbital, which is confined to the fluorobenzene moiety in the *cis* isomer.


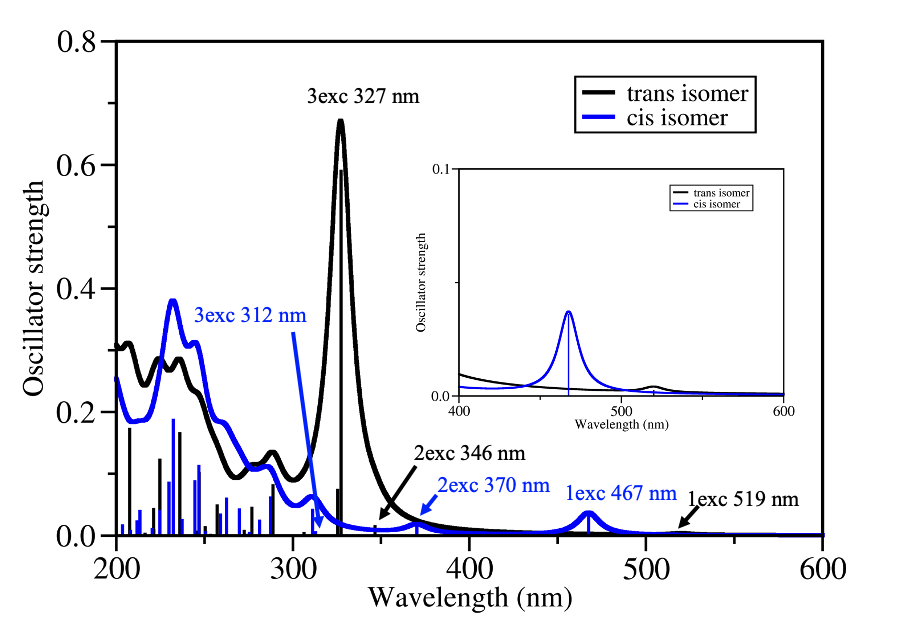


**Figure S3:** Theoretical absorption spectra of the linker molecule (*trans* and *cis* isomer) performed with B3LYP/def2-TZVP in the gas phase. In the inset, the enlarged region between 500-600 nm is depicted. The first three excitations (exc) with the respective wavelength (in nm) are highlighted for both isomers.

**
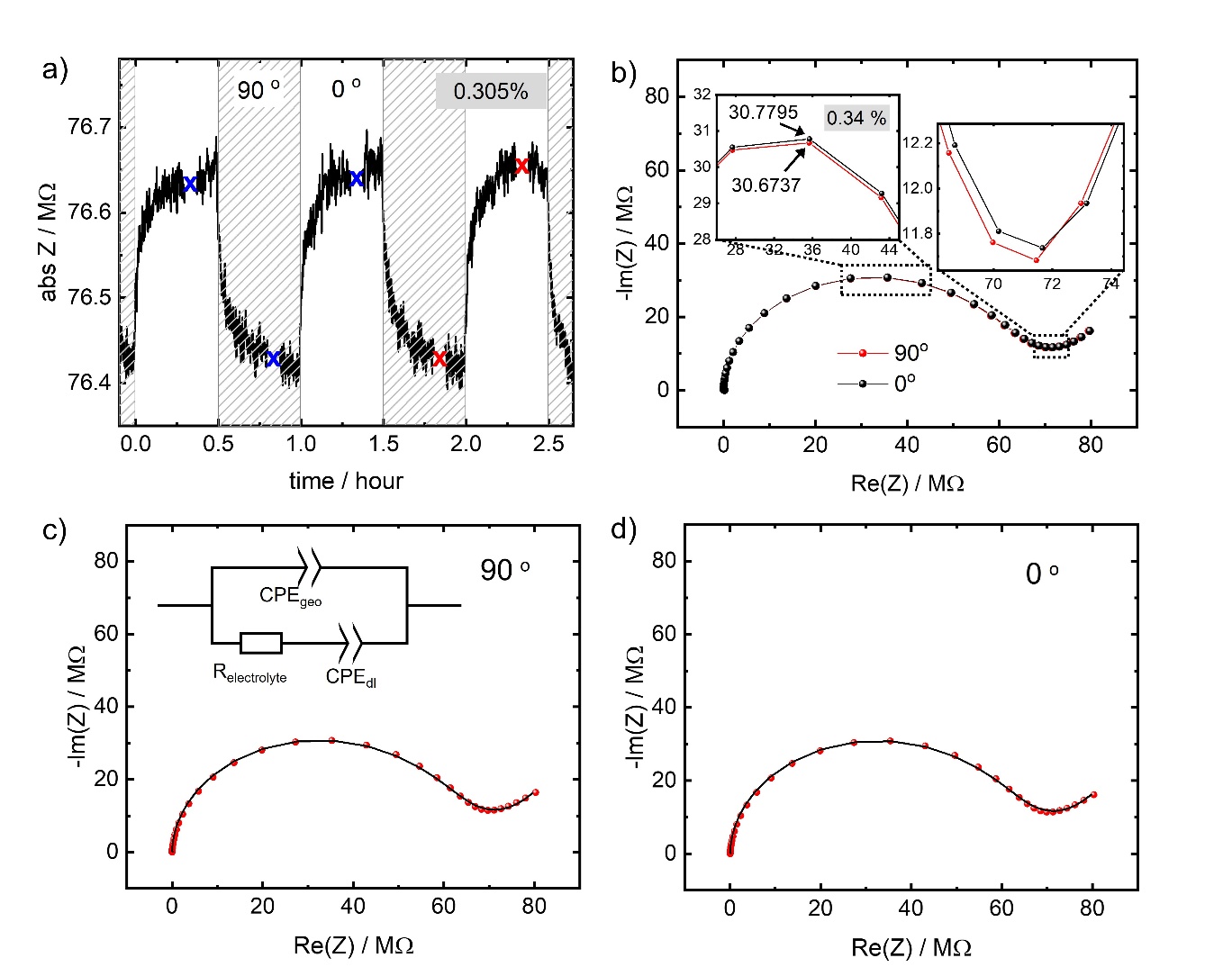
**

**Figure S4:** **a)** and **b)** Absolute value of the impedance *vs*. time curve for different light irradiation angles for the sample in Figure 4 with the corresponding Nyquist plot at the red crosses. **c)** and **d)** Nyquist plots at 0 ^o^ and 90 ^o^ fitted by a simplified equivalent circuit (inset).^[1]^ The fits are the black lines.


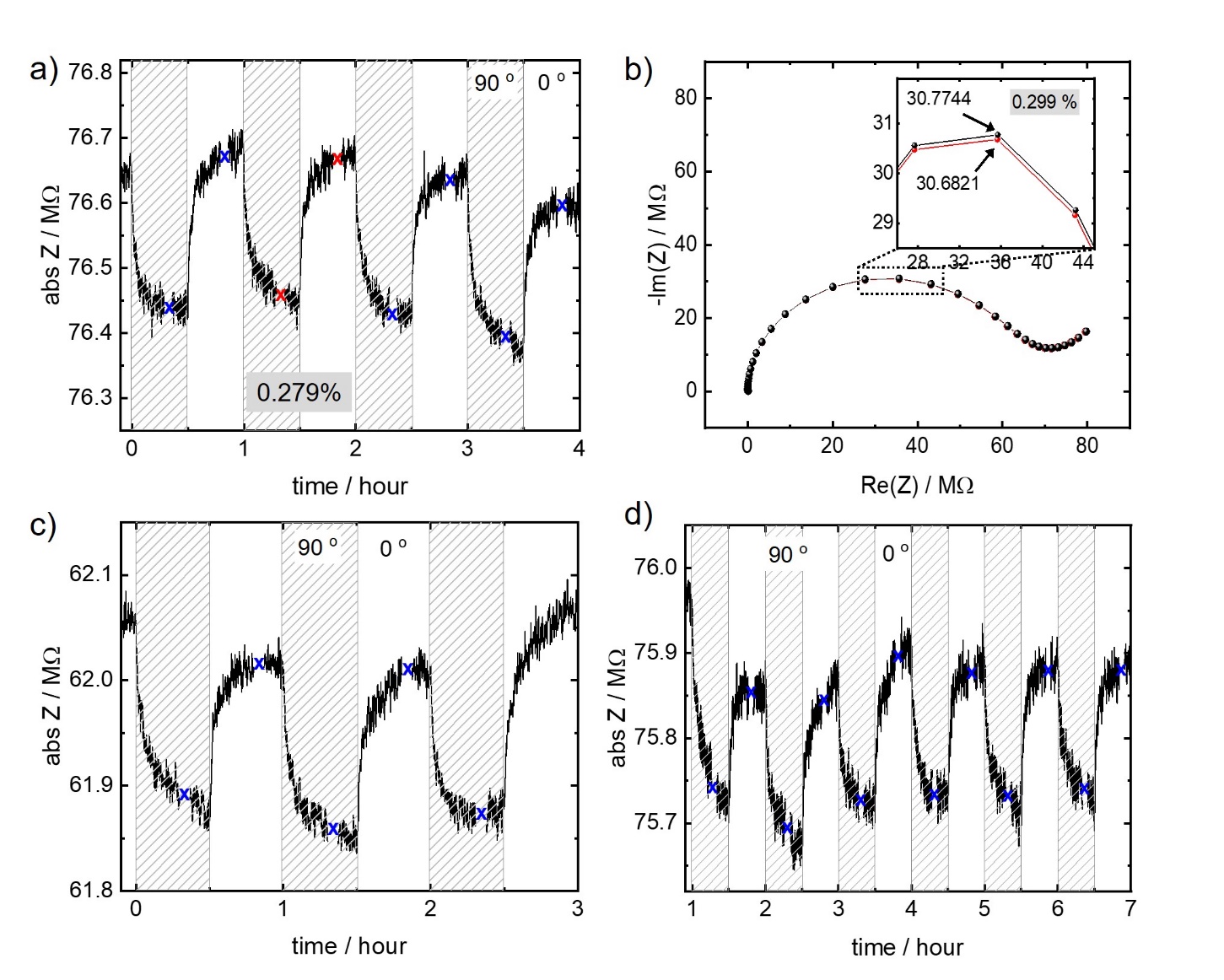
**Figure S5:** **a)** and **b)** impedance *vs.* time and Nyquist plot for a sample prepared in the same way as the sample in Figure 4. **c)** and **d)** impedance *vs*. time curves for samples prepared identically as the sample in Figure 4.


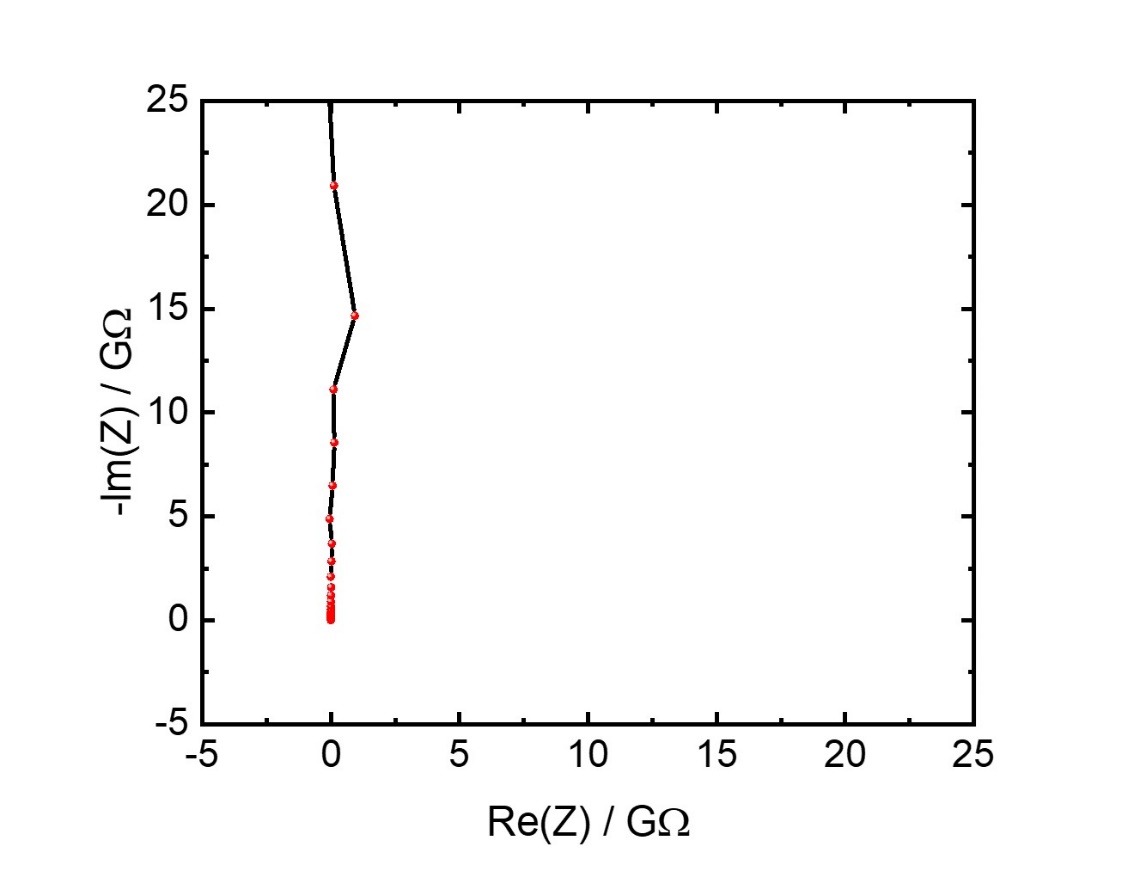


**Figure S6:** Nyquist plot of the empty SURMOF sample.

**
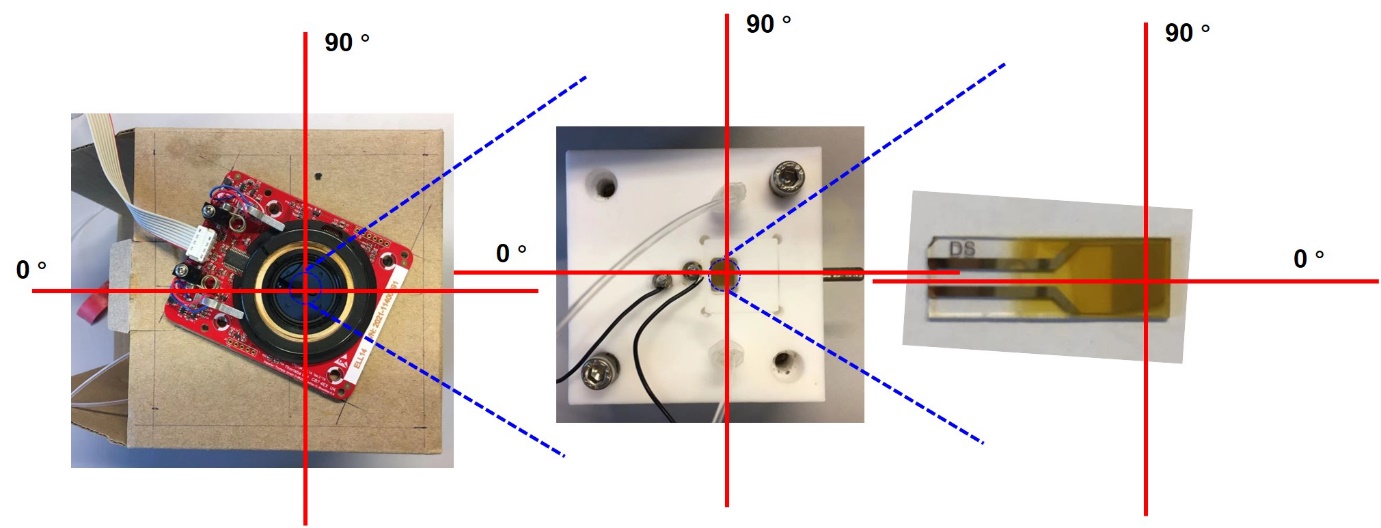

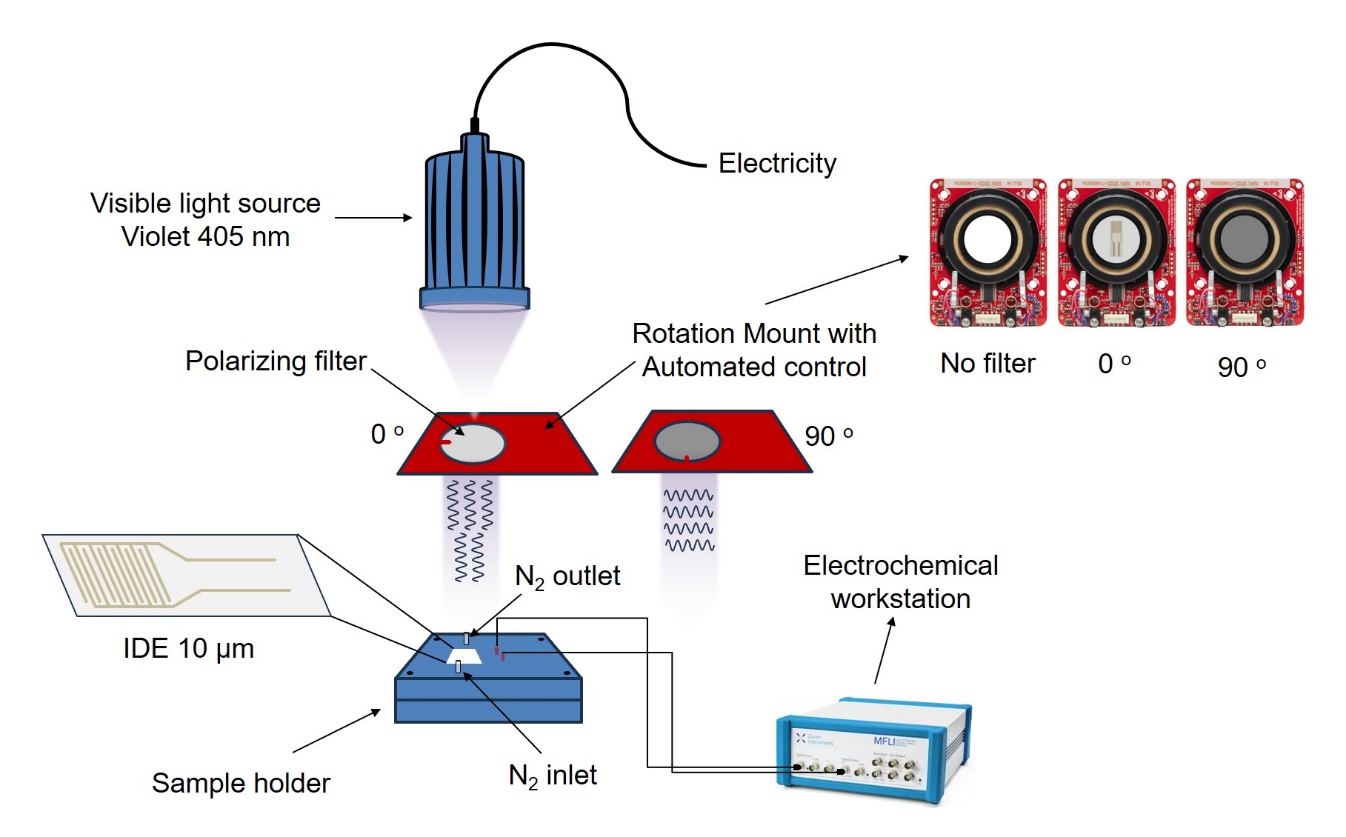
**

**Figure S7:** Sketch of IDE and MOF and LPL.

**Molecular modelling of the Cu(F_2_AzoBDC)(dabco)**

In the Cu(F_2_AzoBDC)(dabco), the DFT-optimized structure^[2]^ has lattice constants of approximately 1 nm, 1 nm and 0.93 nm, while the linear AzoBDC has a length of 1.1 nm. That is, the F_2_AzoBDC cannot be fitted into the MOF if it is fully extended. The DFT-optimized empty MOF structure features staggered π-π stacking in the [100]-linkers and [010]-linkers. The aromatic ring in the Azobenzene couples with neighboring azo groups as well as the aromatic ring in the BDC base, as illustrated in **Figure S8**.


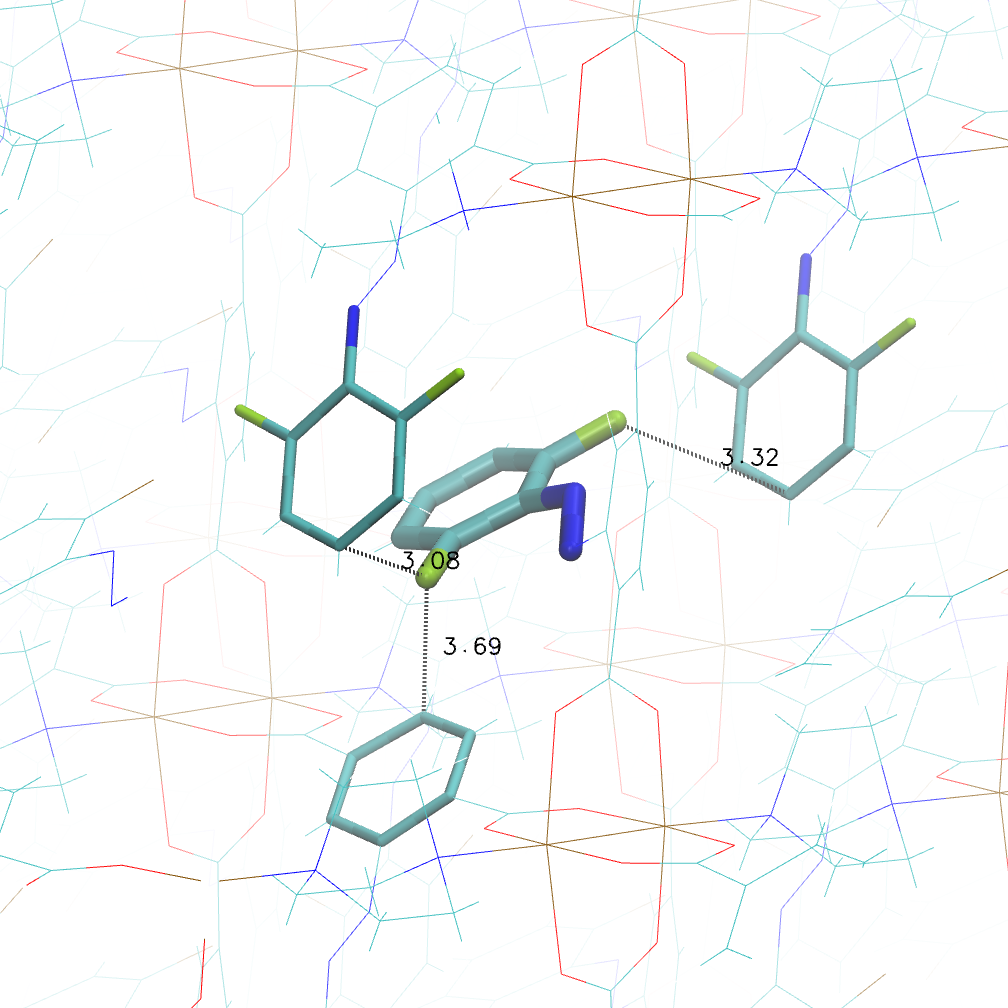


**Figure S8:**  The atomic distances in Angstroms between a [010]-linker (thick) and neighbouring Azo groups and the BDC base (thin). The MOF structure is represented by wireframes. The color codes correspond to chemical elements: cyan for Carbon, blue for Nitrogen, red for Oxygen, green for Fluorine and Brown for Copper. Ring-to-ring distances of less than 0.4 nm corresponds to pronounced π-π stacking.

The preferred residence of Azo groups in the DFT-optimized unit cell also agrees well with the relaxed structure of IL@Cu(F_2_AzoBDC)(dabco). The 5% IL loading corresponds to mostly empty framework with only a few MOF pores filled with ions, which marginally alter the linker stacking.


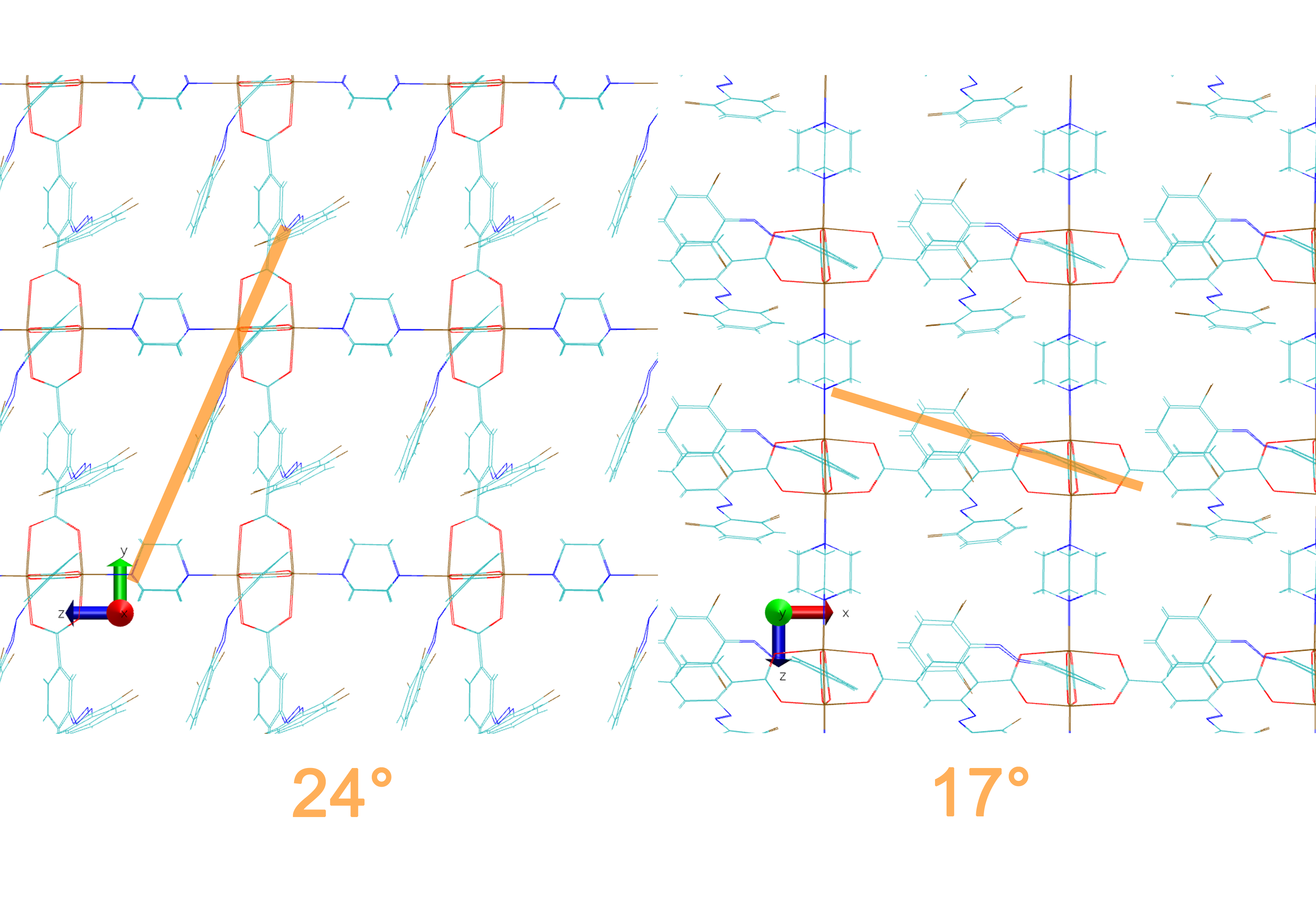


[100] view

[010] view

**Figure S9:** Preferred alignment of the azobenzene in DFT-optimized Cu(F_2_AzoBDC)(dabco) structure. The [100]-linkers and [010]-linkers stack with 24° and 17° off the *xy*-planes.

In the “natural configuration”, both DFT-optimized structure for the empty MOF^[2]^ and the force-field model of low loading IL@Cu(F_2_AzoBDC)(dabco) consistently show anisotropic linker stacking, in **Figure S9** and **S10**. However, in the experiment, the ~27° vs 17° anisotropic linker alignment cannot be detected as the SURMOF has many domains. As ILs are added to the MOF, we would always utilize an [100]-direction electric field to drive the ion transport, thus excluding the anisotropy from the initial alignment for [100]- (*x*-) and [010]- (*y*-) linkers. As the IL flow is switched on by the *E*-field, ILs make impact with the F_2_AzoBDC linker and the average linker alignment is taken from snapshots as in **Figure S10**. The time-evolution of average rotational angle with respect to the *xy*-plane for either *x*- or *y*-linkers are shown in **Figure S11**.

The *x*-linkers are locked at variable rotational angles of 42°, 35°, and 32° corresponding to rotation bias strengths of 4, 10, and 40 kcal/mol. There, the *x*-linkers face their sides against the IL flow, while the ever more rigid locking of linker alignment hinders the ion flow. In contrast, a stronger bias in the *y*-linker moves the F_2_AzoBDC away from the pore, to result in promoted IL flows, in **Table S2**.

**Figure S10:** Force-field modelling of IL@Cu(F_2_AzoBDC)(dabco), before and after forced rotation with 40 kcal/mol bias in the C-C-C-O dihedral in the BDC base, and switching on the electric field. The linker alignments before and after application of the bias are marked by orange reference lines which correspond to the steady state in the time series data in **Figure S11**.


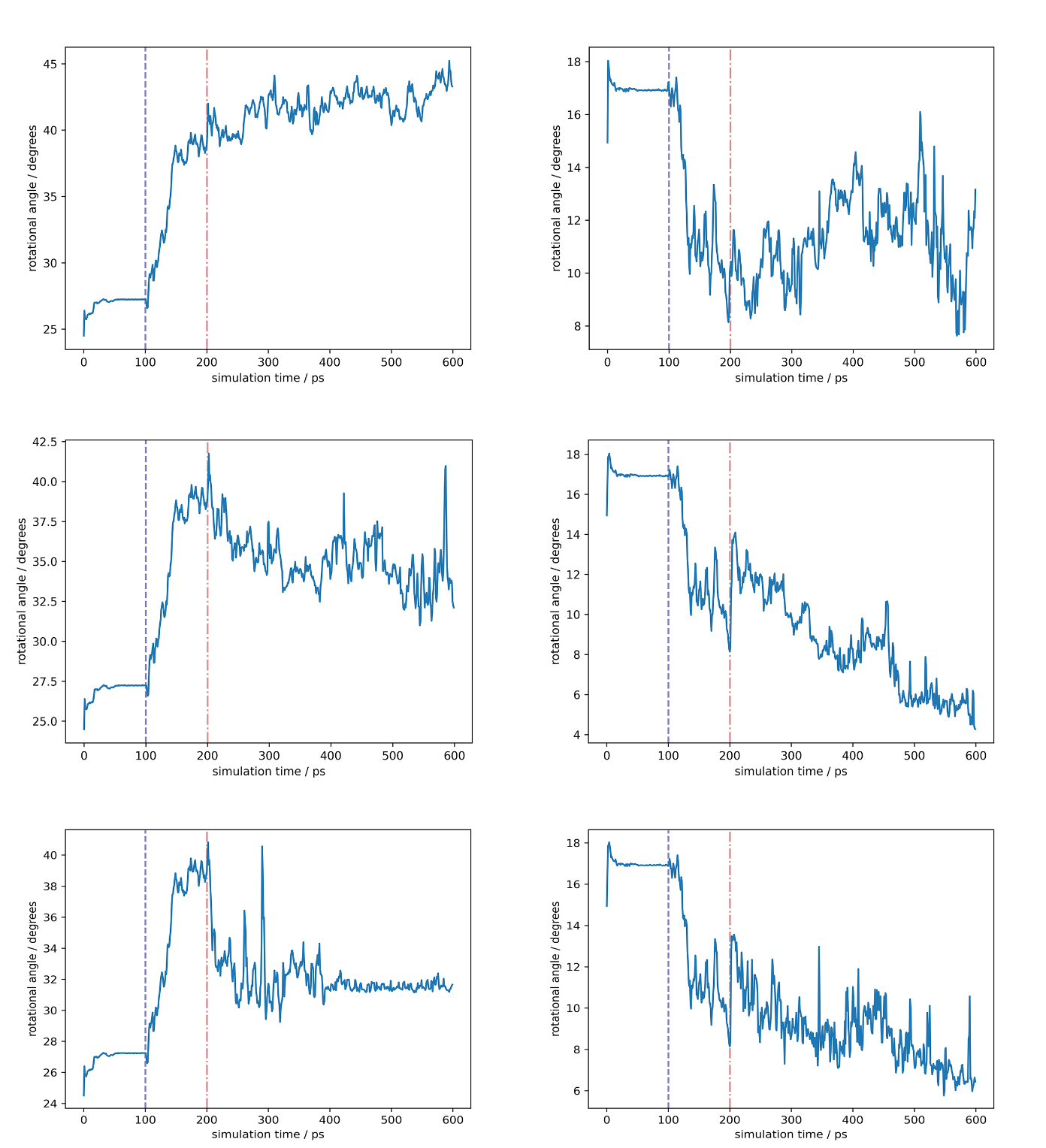


4 kcal/mol bias,

X-linker

10 kcal/mol bias,

X-linker

40 kcal/mol bias,

X-linker

40 kcal/mol bias,

Y-linker

10 kcal/mol bias,

Y-linker

4 kcal/mol bias,

Y-linker

**Figure S11:** Time evolution of rotational angles before the application in the “natural configuration” (before blue dashed lines), during linear IL transport under an external *E*-field (between blue dashed lines and red dash-dot lines), and “locked” states under artificial bias which rotates the F_2_AzoBDC. For Y-linkers, as stronger biases are applied, the linkers are moved away from the IL flow.

| **Bias strength (kcal/mol)** | **0 (dark)** | **4** | **10** | **40** |
| --- | --- | --- | --- | --- |
| IL mobility under X-bias | 1.29 | 1.24 | 0.52 | 0.46 |
| IL mobility under Y-bias | 1.29 | 1.46 | 1.51 | 1.67 |
| Relative mobility w/ X-bias | 100.0% | 96.1% | 40.3% | 35.4% |
| Relative mobility w/ Y-bias | 100.0% | 113.8% | 117.6% | 130.0% |

**Table S2:** IL mobility under either [100]-bias or [010]-bias. A stronger bias strength on the *x*-linkers always reduce the IL mobility while stronger bias promotes ion flow when applied to *y*-linkers.


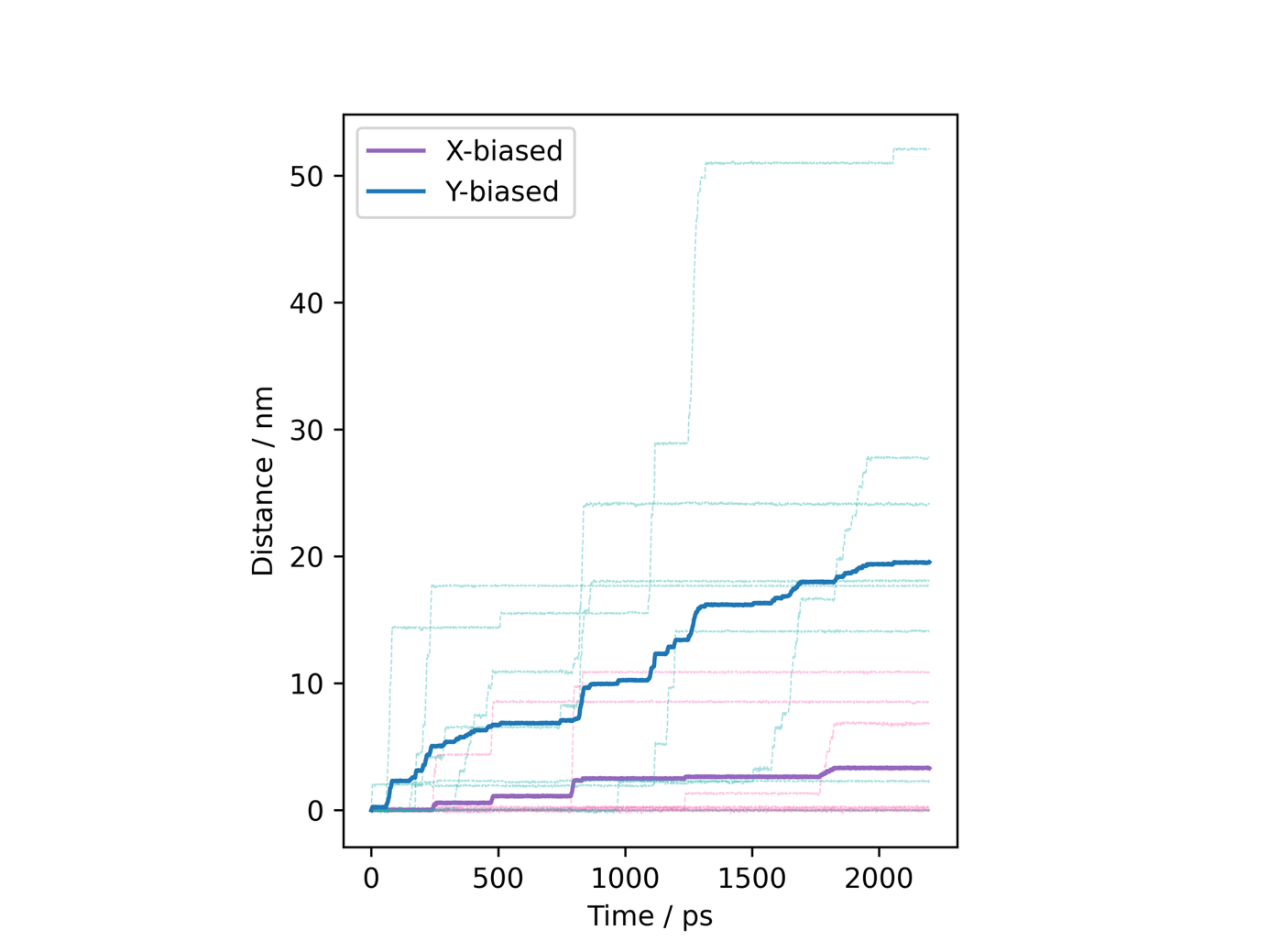


**Figure S12.** Simulated aligned MOF structure with the alignment bias along the *y*-direction ([010] direction), see Figure 5. The travelled distance of the ions as function of simulation time for the MOF aligned in different directions, see legend. The data from the individual trajectories (thin lines) and the average (thick lines) are shown. The travelled IL distance in the *y-*biased MOF (blue; i.e. mimicking LPL with [010] polarization, that is perpendicular to the charge transport direction) is approximately 6 times larger than the travelled IL distance in the *x-*biased MOF (violet; i.e. mimicking LPL with [100] polarization, that is parallel to the charge transport direction). Figure 5 shows a zoom in of the data.


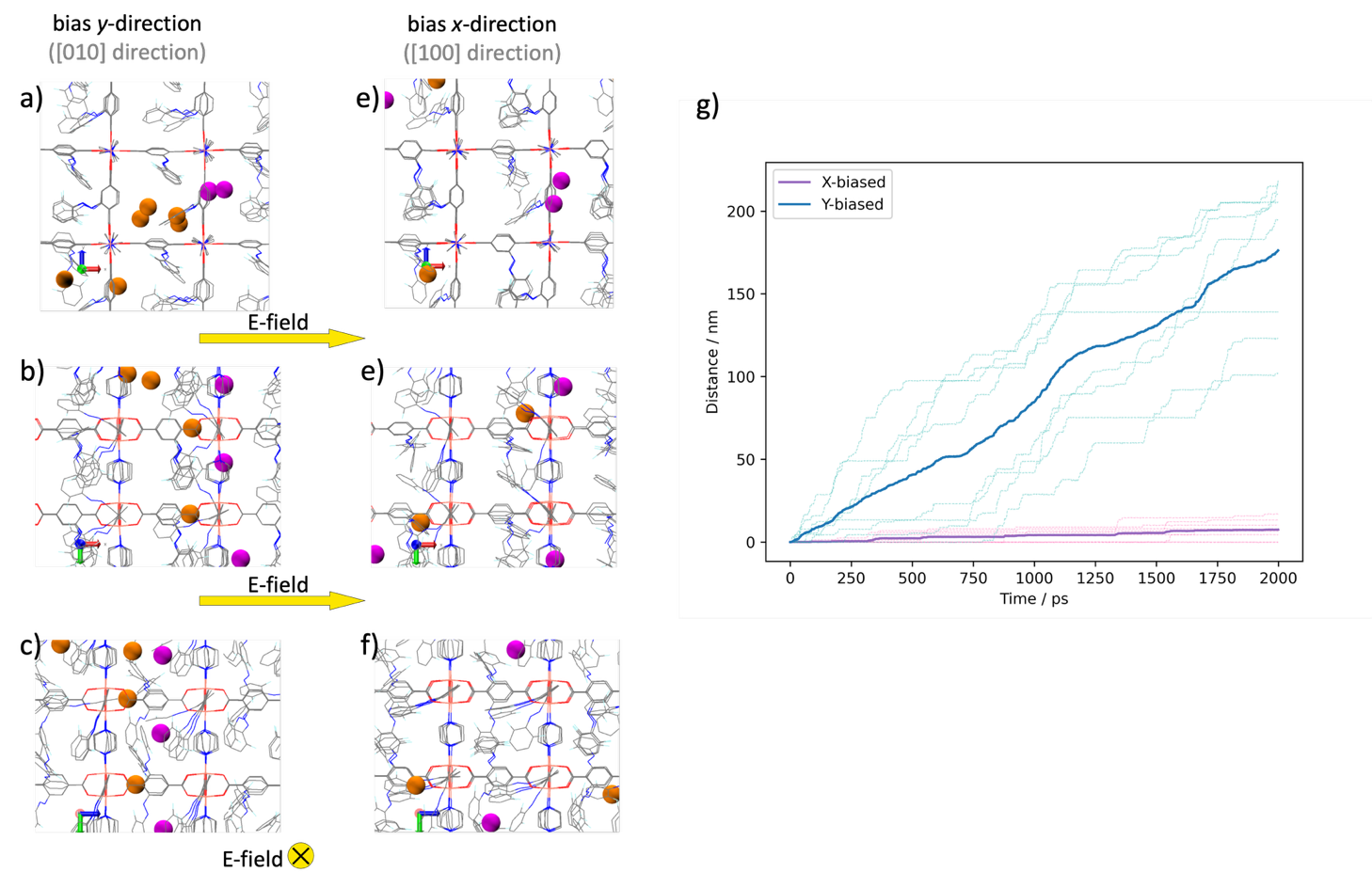


**Figure S13.** Simulated aligned MOF structure with the alignment bias along the *y*-direction ([010] direction) shown in **a-c)** and along the *y*-direction ([010] direction) in **d-f)**, similar to Figure 5. The alignment (see text and methods) mimics the photoalignment by LPL, which is indicated by the violet arrows, in analogy to Figure 1b. (This means, the light propagates in [001] direction where the light is polarized in [010] direction (left, a-c) and in [100] direction (right, d-f).) Spontaneous stacking of the linker moieties within MOF pores are accentuated by thickened azo groups. The view directions on the MOF structure is along [001] in **a)** and **d)**, [010] direction in **b)** and **e)**, and [100] direction in **c)** and **f)**. See also tripods, which indicate the crystal directions like in Figure 1b; [100] - red, [010] - blue and [001] – green. The ion transport, cation in orange and anion in magenta, through the MOF is driven by an electric field in [100] direction, marked by the yellow arrows. The ions have a similar size and mass like oxygen. **g)** The travelled distance of the ions as function of simulation time for the MOF aligned in different directions, see legend. The data from the individual trajectories (thin lines) and the average (thick lines) are shown. The travelled IL distance in the Y-biased MOF (i.e. mimicking LPL with [010] polarization, that is perpendicular to the charge transport direction) is approximately 20 times larger than the travelled IL distance in the X-biased MOF (i.e. mimicking LPL with [100] polarization, that is parallel to the charge transport direction).

**Unlike in Figure 5, the transport of small round ions (rather than relatively large ions of [BMIM][TFSI]) are considered here.**

**Figure S14.** XRD of the Cu_2_(F_2_AzoBDC)_2_(dabco) MOF powder. Experimental data (black) is compared with the calculated diffractogram of the targeted structure (grey). The data are recorded with an X-ray wavelength of 0.154 nm.

**References:**

[1] A. Chandresh, Z. J. Zhang, L. Heinke, Insights in the Ionic Conduction inside Nanoporous Metal-Organic Frameworks by Using an Appropriate Equivalent Circuit, *Materials* **2021**, *14*, 4352.

[2] A. B. Kanj, J. Bürck, N. Vankova, C. Li, D. Mutruc, A. Chandresh, S. Hecht, T. Heine, L. Heinke, Chirality Remote Control in Nanoporous Materials by Circularly Polarized Light, *Journal of the American Chemical Society* **2021**, *143*, 7059-7068.
